# Prognostic and clinicopathological value of dbc1 expression in human cancers: a systematic review and meta-analysis

**DOI:** 10.3389/fonc.2025.1584622

**Published:** 2025-07-07

**Authors:** Haojia Wang, Xinhong Cheng, Bruce Xianzhuo Zhang, Yong Wang, Shuo Gao, Fanghui Ding, Xiaojing Song, Dandan Li, Haixu Ni, Yang Luo, Xun Li

**Affiliations:** ^1^ The First School of Clinical Medicine, Lanzhou University, Lanzhou, China; ^2^ Department of General Surgery, The First Hospital of Lanzhou University, Lanzhou, China; ^3^ National Clinical Key Specialty of General Surgery, The First Hospital of Lanzhou University, Lanzhou, China; ^4^ Cancer Prevention and Treatment Center of Lanzhou University School of Medicine, Lanzhou, China; ^5^ Precision Medicine Laboratory, The First Hospital of Lanzhou University, Lanzhou, China; ^6^ Key Laboratory Biotherapy and Regenerative Medicine of Gansu Province, Lanzhou, China; ^7^ Hepatopancreatobiliary Surgery Institute of Gansu Province, Lanzhou, China; ^8^ Clinical Research Center for General Surgery of Gansu Province, Lanzhou, China; ^9^ Department of Gastroenterology, The First Hospital of Lanzhou University, Lanzhou, China; ^10^ Gansu Province Clinical Research Center for Digestive Diseases, The First Hospital of Lanzhou University, Lanzhou, China; ^11^ Faculty of Health Sciences, University of Macau, Macau, Macau SAR, China; ^12^ The First Affiliated Hospital, Sun Yat-Sen University, Guangzhou, China

**Keywords:** DBC1, carcinoma, meta-analysis, prognosis, clinicopathological characteristics

## Abstract

**Background:**

DBC1 is a large nuclear protein that is thought to influence the development of several human cancers. However, further research has revealed that the relationship between DBC1 and the prognosis and pathological characteristics of cancer patients is controversial. The aim of this paper is to explore the significance of DBC1 in cancer through the method of meta-analysis.

**Methods:**

A systematic search of the PubMed, Web of Science, Embase, CNKI, and Wanfang databases was conducted, resulting in the identification of 25 studies encompassing 4014 patients. The Hazard Ratio (HR) and ratio ratios (RR) were combined using STATA 14.0 software, and 95% confidence intervals (CI) were obtained to assess the association of DBC1 with prognostic and pathologic characteristics of cancer patients.

**Results:**

Meta-analysis of the combined results demonstrated that patients with cancer who exhibited DBC1 overexpression exhibited shorter overall survival (OS) (n = 17, HR = 1.948, 95%CI: [1.280-2.964], P = 0.002, *I^2^* = 88.6) and recurrence-free survival (RFS) (n = 11, HR = 2.182, 95%CI: [1.430-3.330], P = 0.000, *I^2^* = 87.8) rates. In terms of pathological features, elevated DBC1 expression was indicative of poor TNM stage (n = 23, RR = 1.245, 95%CI: [1.012-1.531], P = 0.038, *I^2^* = 79.3), distant metastasis (n = 11, RR = 1.987, 95%CI: [1.021-3.866], P = 0.043, *I^2^* = 63.8), and histologic grade (n = 18, RR = 1.433, 95%CI: [1.115-1.843], P = 0.005, *I^2^* = 79.2).

**Conclusion:**

DBC1 overexpression is associated with poor survival cycle and pathologic features in cancer patients, and it has the potential to be a predictive prognostic marker for cancer. However, more high-quality prospective studies are still needed to validate our conclusions.

**Systematic review registration:**

https://www.crd.york.ac.uk/prospero/, identifier CRD42023426104.

## 1 Introduction

Cancer represents a major global public health challenge, ranking as the leading or second leading cause of death before the age of 70 in 112 countries. Annually, it accounts for approximately 10 million deaths, a figure that continues to rise progressively (1). Despite advancements in diagnostic technologies and early screening methods, the overall burden of cancer on public health remains substantial (2). Surgical intervention continues to serve as a primary treatment modality, even as substantial progress has been achieved in radiotherapy, chemotherapy, and targeted therapies (3). Early-stage cancers are often asymptomatic or present with nonspecific symptoms, resulting in delayed diagnoses. Consequently, many patients present with advanced disease at the time of diagnosis, often rendering surgical treatment infeasible. Early detection and reliable prognosis prediction are therefore essential to enhance treatment outcomes and improve cancer management strategies. Pathological features such as TNM stage, lymphatic infiltration, distant metastasis, and vascular invasion are currently regarded as critical factors influencing patient prognosis. However, these conventional markers increasingly fall short of clinical demands, particularly in accounting for individual patient variability. Their limited efficacy in guiding personalized treatment highlights the urgent need for more precise and sensitive tumor biomarkers. The discovery and development of such biomarkers have become a pivotal focus in modern oncological research and clinical practice.

In 2002, Hamaguchi et al. (4) identified a deletion on chromosome 8p21 in 3.5% of breast cancer patients (7 out of 200 cases), which led to the cloning of Deleted in Breast Cancer 1 (DBC1). This gene, also known as Cell Cycle and Apoptosis Regulator 2 (CCAR2) due to its structural and functional similarity to CCAR1. The DBC1 protein is a large nuclear protein containing multiple structural domains, including S1-like, NL, LZ, EF-Hand, and CC domains (5). These domains facilitate extensive protein-protein interactions, enabling DBC1 to fulfill critical physiological functions (6). One of the principal functions of DBC1 is its interaction with SIRT1, a deacetylase, where it acts as an endogenous inhibitor. This inhibition is mediated through the leucine zipper (LZ) domain of DBC1, which binds directly to SIRT1, forming the DBC1/SIRT1 complex (7). The complex suppresses SIRT1’s deacetylase activity, thereby modulating the activity of downstream targets, including pivotal proteins such as P53 and FOXO. This regulation plays a significant role in various physiological and pathological processes, including tumorigenesis. Beyond its effects on P53 and FOXO, the DBC1/SIRT1 complex also regulates FOXP3 and RORγt, two transcription factors involved in the development of Th17 and Treg cells. By regulating the Th17/Treg balance, DBC1/SIRT1 contributes to the modulation of inflammatory responses in animal models (8). The downstream protein P53, a key tumor suppressor, has been increasingly implicated in cancer biology. The DBC1-SIRT1-P53 axis has emerged as a critical signaling pathway in the regulation of tumorigenesis, with its dysregulation linked to cancer progression (9, 10). Current research highlights the potential of DBC1 as a novel biomarker for predicting cancer occurrence and prognosis. These findings underscore its potential utility in advancing personalized oncology and improving clinical outcomes.

Numerous studies have reported an association between DBC1 overexpression and poor prognosis across various cancers, including gastric cancer (11–13), breast cancer (14, 15), soft tissue sarcoma (16) colon cancer (17, 18), ovarian cancer (19) and small cell lung cancer (20, 21). Adverse clinical outcomes linked to DBC1 overexpression include reduced overall survival (OS) and recurrence-free survival (RFS), advanced TNM stage, increased lymph node infiltration, and higher rates of distant metastasis. In contrast, other studies have reported a positive association between DBC1 overexpression and improved prognosis in certain cancer types (22, 23). These contradictory results the complexity of the relationship between DBC1 expression and cancer progression, highlighting the need for further investigation. To resolve these inconsistencies, a comprehensive synthesis of the current evidence is necessary. Several studies have previously explored the association between DBC1 expression and cancer. For instance, Liu et al. (24). emphasized its prognostic relevance in digestive system malignancies, while another meta-analysis published in 2019 examined the relationship between DBC1 expression and patient survival outcomes (25). However, no study has systematically evaluated DBC1 expression in human cancers and its impact on patient prognosis and clinicopathological characteristics. Furthermore, the data included in these earlier studies are relatively outdated, lacking the integration of findings from the past decade, which limits their relevance to contemporary oncological practice. This study aims to systematically evaluate the prognostic and clinicopathological implications of DBC1 expression across multiple human cancer types, focusing specifically on studies assessing DBC1 in primary tumor tissues and reporting quantifiable survival outcomes or pathological parameters. By excluding non-tissue-based studies, non-clinical reports, and data-limited publications, we aim to ensure clinical relevance, methodological consistency, and statistical rigor. Meta-analysis, as a robust statistical tool, enables integration of heterogeneous findings and provides more reliable estimates of the true prognostic value of DBC1. Our results may offer novel insight into the clinical potential of DBC1 as a prognostic biomarker, with possible implications for early cancer detection, risk stratification, and treatment monitoring.

## 2 Methods

This study is registered with PROSPERO (registration number CRD42023426104) and has been approved by all contributing authors. We used all PRISMA (Preferred Reporting Items for Systematic Reviews and Meta-Analyses) terms and followed the PRISMA checklist to complete the meta-analysis (26). The research question formulated based on the PICOS principles is: “Does overexpression of DBC1 predict the prognosis and pathologic features of cancer patients? “ The population “P” was defined as patients with pathologically confirmed malignant cancer, the intervening factor “I” was DBC1 overexpression, and the control factor “C” was normal DBC1 expression. The indicator “O” represents prognostic and pathologic characteristics. Prognostic indicators included overall survival (OS), disease-free survival (DFS), and recurrence-free survival (RFS). In this study, DFS and RFS were combined since they have similar meanings. For study type “S”, we selected randomized controlled trials, case-control studies, and cohort studies.

### 2.1 Search strategy

A systematic literature search was conducted in PubMed, Web of Science, Embase, CNKI, and Wanfang databases, covering publications up to May 11, 2023. The search was restricted to studies published in English or Chinese and included only peer-reviewed journal articles. Grey literature, such as conference abstracts, dissertations, and unpublished studies, was excluded due to insufficient methodological detail, limited availability of extractable data, and the absence of peer review, which may compromise the reliability and quality assessment of the included evidence. We conducted the search using the following keywords and Boolean operators.: “DBC1” OR “deleted in breast cancer 1” OR “DBC-1 “OR “P30DBC “OR “DBIRD Complex Subunit KIAA1967 “OR “CCAR2 “OR “CCAR2 protein” OR “Cell Division Cycle And Apoptosis Regulator Protein 2”. Given the extensive range of cancer-related synonyms, manual screening was performed to ensure comprehensive identification of relevant literature and to minimize the risk of omissions. The detailed search strategy is outlined in **Supplementary Table S1**. Additionally, references from key articles were reviewed to further supplement the dataset and capture any studies potentially overlooked during the database search.

### 2.2 Inclusion and exclusion criteria

This study included articles that met the following criteria: (i) patients with pathologically confirmed malignancies; (ii) assessment of DBC1 expression in primary tumor tissues; (iii) explicit reporting of the detection method, including the scoring system used (e.g., staining cells percentage, staining intensity score, or percentage of positive); (iv) clear definition of DBC1 positivity or cut-off values used to distinguish between high and low expression levels; (v) analysis of the relationship between DBC1 expression and clinical outcomes or clinicopathological characteristics; (vi) availability of sufficient data to calculate hazard ratios (HR) or relative ratios (RR) along with corresponding 95%CI.

Exclusion criteria included: (i) non-human or *in vitro* studies; (ii) reviews, conference abstracts, letters, and case reports; (iii) publications in languages other than English or Chinese; (iv) insufficient statistical data to estimate HR or RR; (v) lack of access to the full text; (vi) duplicate publications; (vii) absence of original clinical data or unclear DBC1 expression definitions.

### 2.3 Data extraction and quality assessment

The studies included in this analysis were independently screened according to predefined selection criteria, and data were extracted using a standardized extraction form. Discrepancies arising during screening and data extraction were resolved through discussion to reach consensus. Extracted data included: first author, year of publication, country, cancer type, number of patients, age, gender, recruitment time, DBC1 detection method, follow-up period, DBC1 expression cases, DBC1 Cutoff Value, survival analysis, HR or RR with 95% CI, and pathologic characteristics. Study quality was assessed using the Newcastle-Ottawa Scale (NOS), a standardized tool that evaluates studies across three domains: selection (0–4 points), comparability (0–2 points), and outcome assessment (0–3 points), with a total score range of 0 to 9. Studies achieving an NOS score greater than 6 were classified as high quality (27).

### 2.4 Statistical analysis

Data analysis was performed using STATA version 14.0, and meta-analytic methods were applied to synthesize prognostic indicators across included studies. The association between DBC1 expression levels and patient prognosis was evaluated using HR with corresponding 95% CI, while RR with 95% CI was used to analyze the relationship between DBC1 expression and clinicopathological characteristics. High DBC1 expression was deemed indicative of a better prognosis if the pooled HR was <1, whereas an HR >1 suggested a poorer prognosis. Results were regarded as not statistically significant if the 95% CI included the null value (HR = 1). Heterogeneity among studies was assessed using Cochrane’s Q test and Higgins’ I² statistic. Significant heterogeneity was defined as P < 0.10 or I² > 50% (28), in which case a random-effects model was employed. In the absence of significant heterogeneity, a fixed-effects model was applied. Subgroup analysis, meta-regression, and sensitivity analysis were conducted to further explore sources of heterogeneity. Publication bias was evaluated using Begg’s and Egger’s tests, complemented by funnel plot visualization (29). Statistical significance was defined as P < 0.05.

## 3 Results

### 3.1 Search results

**Figure 1:**
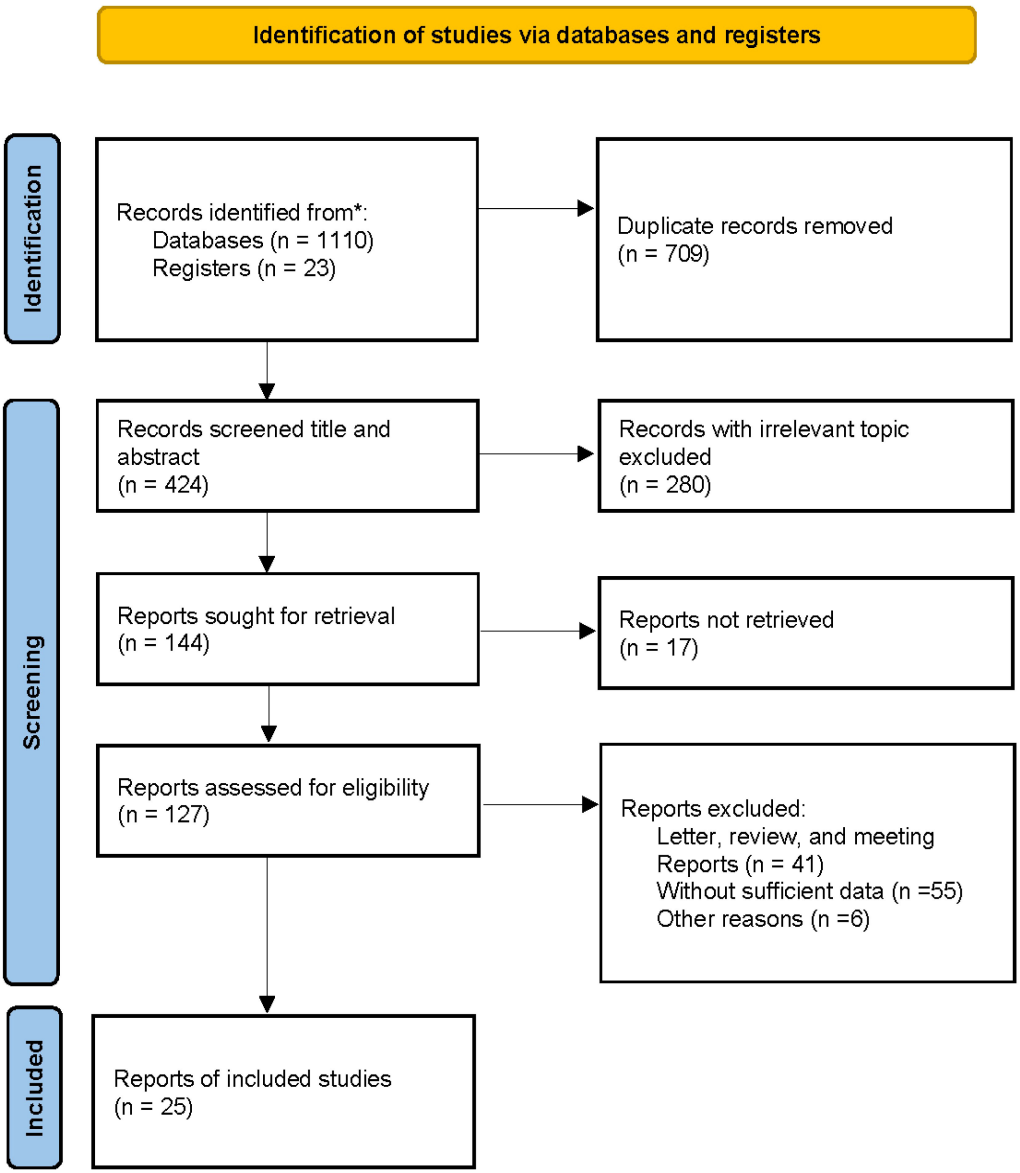
The flow diagram of study selection and exclusion process in the meta-analysis.

A total of 1100 articles were identified through database searches, including 223 from PubMed, 369 from Web of Science, 297 from Embase, 82 from CNKI, and 139 from Wanfang. Additionally, 23 articles were identified through reference lists, resulting in 1133 publications subjected to initial screening. After removing 709 duplicate records, the titles and abstracts of 424 studies were reviewed. Of these, 280 articles were excluded for being irrelevant to the study topic, and a further 119 were excluded as they comprised letters, reviews, conference papers, or studies with insufficient data. Ultimately, 25 articles met the inclusion criteria and were included in the meta-analysis. The detailed screening process is outlined in **Figure 1**.

The characteristics of the included studies are summarized in **Table 1**. The publications, dated from 2009 to 2022, encompass a total of 4014 patients across studies conducted in China, Korea, and Japan. Sample sizes varied between 28 and 557 participants. The included studies involved various types of cancer, including gastric cancer (GC, n = 5) (11–13, 22, 23), breast cancer (BC, n = 2) (14, 15), colorectal cancer (CC, n = 3) (17, 18, 30), hepatocellular carcinoma (HCC, n = 3) (31–33), small cell lung cancer (SCLC, n = 2) (20, 21), esophageal squamous cell carcinoma (ESCC, n = 1) (34), soft tissue sarcomas (n = 1) (16), clear cell renal cell carcinoma (CRCC, n = 1) (35), diffuse large B cell lymphoma (DLBCL, n = 1) (36), ovarian carcinoma (OC, n = 1) (19), osteosarcoma (n = 1) (37), gallbladder carcinoma (n = 1) (38), head and neck squamous cell carcinoma (HNSCC, n = 1) (39), papillary thyroid carcinoma (PTC, n = 1) (40) and endometrial carcinoma (EC, n = 1) (41). A total of 17 studies reported OS, with 12 providing data from univariable analyses and 16 from multivariable analyses. RFS was analyzed in 11 studies, including 9 using univariable analysis and 10 using multivariable analysis. Among the included studies, DBC1 expression was predominantly measured by immunohistochemistry in 24 studies, while one study utilized PCR. The cutoff values for DBC1 expression varied across studies. To standardize this variation and enable subgroup analyses, the cutoff values were categorized into four groups: staining cells ≥ 30% (n = 6), SI × PP ≥ 7 (n = 7), SI + PP ≥ 6 (n = 3), and Other (n = 9) (**Table 2**). The quality of the included studies was assessed using the Newcastle-Ottawa Scale (NOS), with all 25 studies scoring between 6 and 9, indicating high methodological quality.

**Table 1:** Characteristics of the included studies.

References	Country	Type of Cancer	Patient	Age (years)	Male/Female	Recruitment time	Follow-up period	Outcomes	HR (95% CI)	Quality score
Cha (11)	Korea	GC	177	< 60: 53 ≥ 60: 124	135/42	1997-2005	Up to 2008.3	OS (U) RFS (U) OS (M) RFS (M)	3.914 (2.044-7.494) 3.45 (1.923-6.187) 3.334 (1.557-7.139) 2.321 (1.182-4.558)	8
Lee (14)	Korea	BC	122	< 50: 85 ≥ 50: 37	NA	1997-2002	Up to 2008.3	OS (U) RFS (U) OS (M) RFS (M)	13.875 (1.892-101.769) 4.559 (1.799-11.553) 14.12 (1.913-104.211) 3.865 (1.514-9.868)	9
Kang (22)	Korea	GC	452	< 60: 213 ≥ 60: 239	309/143	2002-2005	53.3 (3–83) months	OS (M)	0.581 (0.381-0.886)	8
Kim (34)	Korea	ESCC	165	< 65: 38 ≥ 65: 127	159/6	NA	NA	OS (U) RFS (U) OS (M) RFS (M)	3.142 (1.890-5.222) 2.889 (1.822-4.582) 2.830 (1.680~4.767) 2.655 (1.650~4.274)	8
Jung (30)	Korea	CC	349	< 65: 186 ≥ 65: 163	208/141	2002-2009	55.3 months	OS (U)	1.396 (0.818-2.381)	7
Kim (16)	Korea	Soft tissue sarcomas	104	< 60: 67 ≥ 60: 37	59/45	1998-2011	Up to 2012.10	OS (U) OS (M)	2.338 (1.090–5.013) 6.501 (2.160–19.565)	8
Noh (35)	Korea	CRCC	200	≤ 55: 82 > 55: 118	140/60	1998-2011	Up to 2011.12	OS (U) RFS (U) OS (M) RFS (M)	22.748 (3.087–167.615) 3.030 (1.387–6.617) 26.670 (3.536–201.142) 3.791 (1.634–8.791)	8
Park (36)	Korea	DLBCL	101	< 60: 51 ≥ 60: 50	58/43	1995-2007	61.5 (1–191) months	OS (U) RFS (U) OS (M) RFS (M)	4.699 (1.867–11.827) 4.817 (2.065–11.239) 3.137 (1.230–8.000) 3.909 (1.536–9.950)	8
Noguchi (23)	Japan	GC	557	< 65: 319 ≥ 65: 238	391/166	1999-2002	69 (6-142) months	Clinicopathologic data	NA	6
Zhang (17)	China	CC	186	< 60: 87 ≥ 60: 99	126/60	2004-2006	At least 5 years	OS (U) OS (M)	4.08 (1.76–9.48) 3.44 (1.36–8.69)	9
Cho (19)	Korea	OC	104	< 60: 71 ≥ 60: 33	NA	1996-2008	Up to 2013.8	OS (U) RFS (U) OS (M)	3.474 (1.684-7.166) 3.007 (1.624-5.567) 2.423 (1.144-5.132)	9
Wagle (37)	Korea	Osteosarcoma	35	< 30: 25 ≥ 30: 10	25/10	1998-2012	44 (4-170) months	OS (U) RFS (U) OS (M) RFS (M)	8.639 (1.940–38.469) 6.657 (1.875–23.629) 8.639 (1.940–38.469) 6.555 (1.825–23.549)	8
Won (38)	Korea	Gallbladder carcinoma	104	< 62: 44 > 62: 60	51/53	1982-2009	46.5 (2-274)	OS (M)	0.429 (0.194-0.950)	8
Ha (31)	Korea	HCC	199	≤ 55: 125 > 55 74	161/38	2000-2006	119.1 (24.0-151.4)	OS (U) RFS (U) OS (M) RFS (M)	1.205 (0.627-2.319) 1.789 (1.031-3.102) 1.108 (0.561-2.186) 1.710 (0.969-3.018)	9
Zhang (18)	China	CC	150	< 60: 72 ≥ 60: 78	102/48	2009-2010	60 months	OS (M)	1.67 (1.29-2.14)	8
Li (32)	China	HCC	119	< 60: 59 ≥ 60: 60	74/45	2007-2009	At least 4 years	OS (U) DFS (U) OS (M) DFS (M)	3.425 (2.043-5.742) 2.906 (1.815-4.652) 0.458 (0.254-0.826) 0.601 (0.365-0.990)	7
Wang (12)	China	GC	201	< 60: 94 ≥ 60: 107	143/58	2011-2016	60 months	OS (M) DFS (M)	0.675 (0.471-0.981) 0.694 (0.476-1.059)	6
Zhang (20)	China	SCLC	73	< 60: 37 ≥ 60: 36	59/14	2009-2013	Up to 2017.5	OS (M) DFS (M)	3.529 (2.663-4.667) 2.776 (2.136-2.583)	7
Sung (15)	Korea	BC	28	≤ 52: 17 > 52: 11	NA	2004-2009	NA	Clinicopathologic data	NA	7
Duan (33)	China	HCC	120	< 60: 57 ≥ 60: 63	85/35	2005-2010	NA	Clinicopathologic data	NA	7
Yu (39)	China	HNSCC	120	< 60: 67 ≥ 60: 53	109/11	2009-2011	NA	Clinicopathologic data	NA	7
Huan (13)	China	GC	142	< 60: 52 ≥ 60: 90	91/51	2008-2009	44 (5-60) months	Clinicopathologic data	NA	8
Wang (21)	China	SCLC	76	< 60: 23 ≥ 60: 53	70/6	2018-2019	NA	Clinicopathologic data	NA	6
Zhou (40)	China	PTC	70	< 45: 39 ≥ 45: 31	26/44	2016-2019	NA	Clinicopathologic data	NA	6
Zhao (41)	China	EC	60	> 45: 32 ≤45: 28	NA	2019-2020	NA	Clinicopathologic data	NA	7

HR, hazard ratio; CI, confidence interval; GC, gastric cancer; BC, breast cancer; ESCC, esophageal squamous cell carcinoma; CRCC, clear cell renal cell carcinoma; DLBCL, diffuse large B cell lymphoma; CC, Colorectal Cancer; OC, ovarian carcinoma; HCC, hepatocellular carcinoma; SCLC, small cell lung cancer; HNSCC, head and neck squamous cell carcinoma; PTC, papillary thyroid carcinoma; EC, endometrial carcinoma; OS, overall survival; RFS, recurrence-free survival; U, univariable analysis; M, multivariable analysis; NA, not available.

**Table 2:** Cutoff value for DBC1 in the included studies.

References	Detection method	DBC1 (+/-)	Cutoff value for DBC1	Define
Cha (11)	IHC	109/68	Positive if 30% or more of the tumor cells were stained	Staining cells ≥30%
Lee (14)	IHC	87/35	Positive if 30% or more of the tumor cells were stained	Staining cells ≥30%
Kang (22)	IHC	271/181	Positive if 30% or more of the tumor cells were stained	Staining cells ≥30%
Kim (34)	IHC	90/75	Score = SI × PP SI: 1 (weak staining), 2 (moderately staining), 3 (strong staining), PP: 0 (0%-4%), 1 (5%-25%), 2 (26%-50%), 3 (51%-75%) and 4 (76%-100%) Positive if score ≥ 8	SI × PP ≥7
Jung (30)	IHC	183/166	Positive if 70% or more of the tumor cells were stained	Staining cells ≥30%
Kim (16)	IHC	77/27	Score = SI + PP SI: 0 (no staining), 1 (weak staining), 2 (moderately staining), 3 (strong staining), PP: 0 (no staining cells), 1 (1%), 2 (2%-10%), 3 (11%-33%), 4 (34%-66%), 5 (67%-100%) Positive if score ≥ 6	SI + PP ≥6
Noh (35)	IHC	121/79	Score = SI × PP SI: 0 (no staining), 1 (weak staining), 2 (moderately staining), 3 (strong staining), PP: 0 (0%-10%), 1 (11%-30%), 2 (31%-50%), 3 (51%-100%) Positive if score ≥ 3	Other
Park (36)	IHC	74/27	IHC, Positive if 30% or more of the tumor cells were stained	Staining cells ≥30%
Noguchi (23)	IHC	438/119	IHC, Positive if 10% or more of the tumor cells were stained	Other
Zhang (17)	IHC	79/107	Score = SI × PP SI: 0 (no staining), 1 (weak staining), 2 (moderately staining), 3 (strong staining), PP: 0 (no staining cells), 1 (1%-25%), 2 (26%-50%), 3 (51%-75%), 4 (76%-100%) Positive if score ≥ 9	SI × PP ≥7
Cho (19)	IHC	66/38	Score = SI + PP SI: 0 (no staining), 1 (weak staining), 2 (moderately staining), 3 (strong staining), PP: 0 (no staining cells), 1 (1%), 2 (2%-10%), 3 (11%-33%), 4 (34%-66%), 5 (67%-100%) Positive if score ≥ 7	SI + PP ≥6
Wagle (37)	IHC	17/18	Score = SI + PP SI: 0 (no staining), 1 (weak staining), 2 (moderately staining), 3 (strong staining), PP: 0 (no staining cells), 1 (1%), 2 (2%-10%), 3 (11%-33%), 4 (34%-66%), 5 (67%-100%) Positive if score ≥ 6	SI + PP ≥6
Won (38)	IHC	32/72	Score = SI + PP SI: 0 (no staining), 1 (weak staining), 2 (moderately staining), 3 (strong staining), PP: 0 (no staining cells), 1 (1%-30%), 2 (30%-60%), 3 (61%-100%) Positive if score ≥ 5	Other
Ha (31)	IHC	177/22	Positive if 50% or more of the tumor cells were stained	Staining cells ≥30%
Zhang (18)	IHC	61/89	Score = SI × PP SI: 0 (no staining), 1 (weak staining), 2 (moderately staining), 3 (strong staining), PP: 0 (no staining cells), 1 (1%-25%), 2 (26%-50%), 3 (51%-75%), 4 (76%-100%) Positive if score ≥ 7	SI × PP ≥7
Li (32)	IHC	64/55	Score = SI × PP SI: 0 (no staining), 1 (weak staining), 2 (moderately staining), 3 (strong staining), PP: 0 (no staining cells), 1 (1%-25%), 2 (26%-50%), 3 (51%-75%), 4 (76%-100%) Positive if score ≥ 7	SI × PP ≥7
Wang (12)	IHC	151/50	Score = SI × PP SI: 0 (no staining), 1 (weak staining), 2 (moderately staining), 3 (strong staining), PP: 0 (no staining cells), 1 (1%-30%), 2 (31%-50%), 3 (51%-80%), 4 (81%-100%) Positive if score ≥ 4	Other
Zhang (20)	RT-PCR	57/16	Using 2^-ΔΔCt^ median as the cut-off value, patients were divided into high expression and low expression groups	Other
Sung (15)	IHC	25/3	Staining intensity was quantified with a score ranging from 0 to 3. Each score was calculated according to the following equation: total score = (% cells with intensity of negative [0] × 0) + (% cells with intensity of [1+] × 1) + (% cells with intensity of [2+] × 2) + (% cells with intensity of [3+] × 3)	Other
Duan (33)	IHC	6/114	Score = SI × PP SI: 0 (no staining), 1 (weak staining), 2 (moderately staining), 3 (strong staining), PP: 1 (0%-25%), 2 (26%-50%), 3 (51%-75%), 4 (76%-100%) Positive if score ≥ 9	SI × PP ≥7
Yu (39)	IHC	52/68	Score = SI × PP SI: 0 (no staining), 1 (weak staining), 2 (moderately staining), 3 (strong staining), PP: 0 (0%-9%), 1 (10%-29%), 2 (30%-49%), 3 (50%-74%), 4 (75%-100%) Positive if score ≥ 9	SI × PP ≥7
Huan (13)	IHC	13/129	Score = SI × PP SI: 0 (no staining), 1 (weak staining), 2 (moderately staining), 3 (strong staining), PP: 0 (no staining cells), 1 (1%-25%), 2 (26%-50%), 3 (51%-75%), 4 (76%-100%) Positive if score ≥ 9	SI × PP ≥7
Wang (21)	IHC	58/18	Positive if moderately staining or strong staining	Other
Zhou (40)	IHC	50/20	Score = SI × PP SI: 0 (no staining), 1 (weak staining), 2 (moderately staining), 3 (strong staining), PP: 0 (0%-4%), 1 (5%-25%), 2 (26%-50%), 3 (51%-75%) and 4 (76%-100%) Positive if score ≥ 1	Other
Zhao (41)	IHC	42/18	Score = SI + PP SI: 0 (no staining), 1 (weak staining), 2 (moderately staining), 3 (strong staining), PP: 0 (0%-4%), 1 (5%-25%), 2 (26%-50%), 3 (51%-75%) and 4 (76%-100%) Positive if score ≥ 4	Other

DBC1, deleted in breast cancer 1; IHC, immunohistochemistry; SI, staining intensity; PP, percentage of positive.

### 3.2 Correlation between DBC1 expression and prognosis

**Figure 2:**
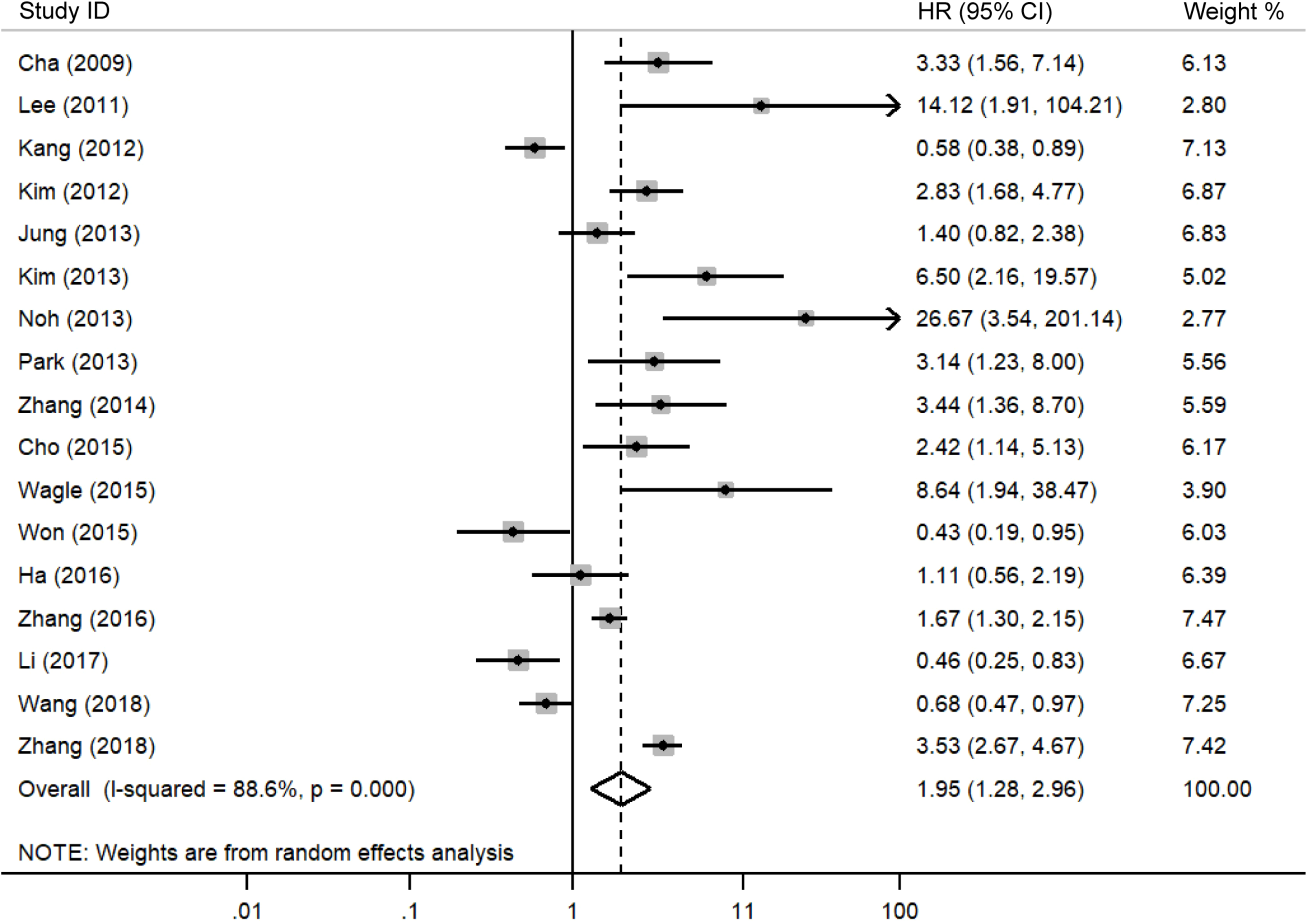
Forest plot of DBC1 expression and OS in various cancers.

**Figure 3:**
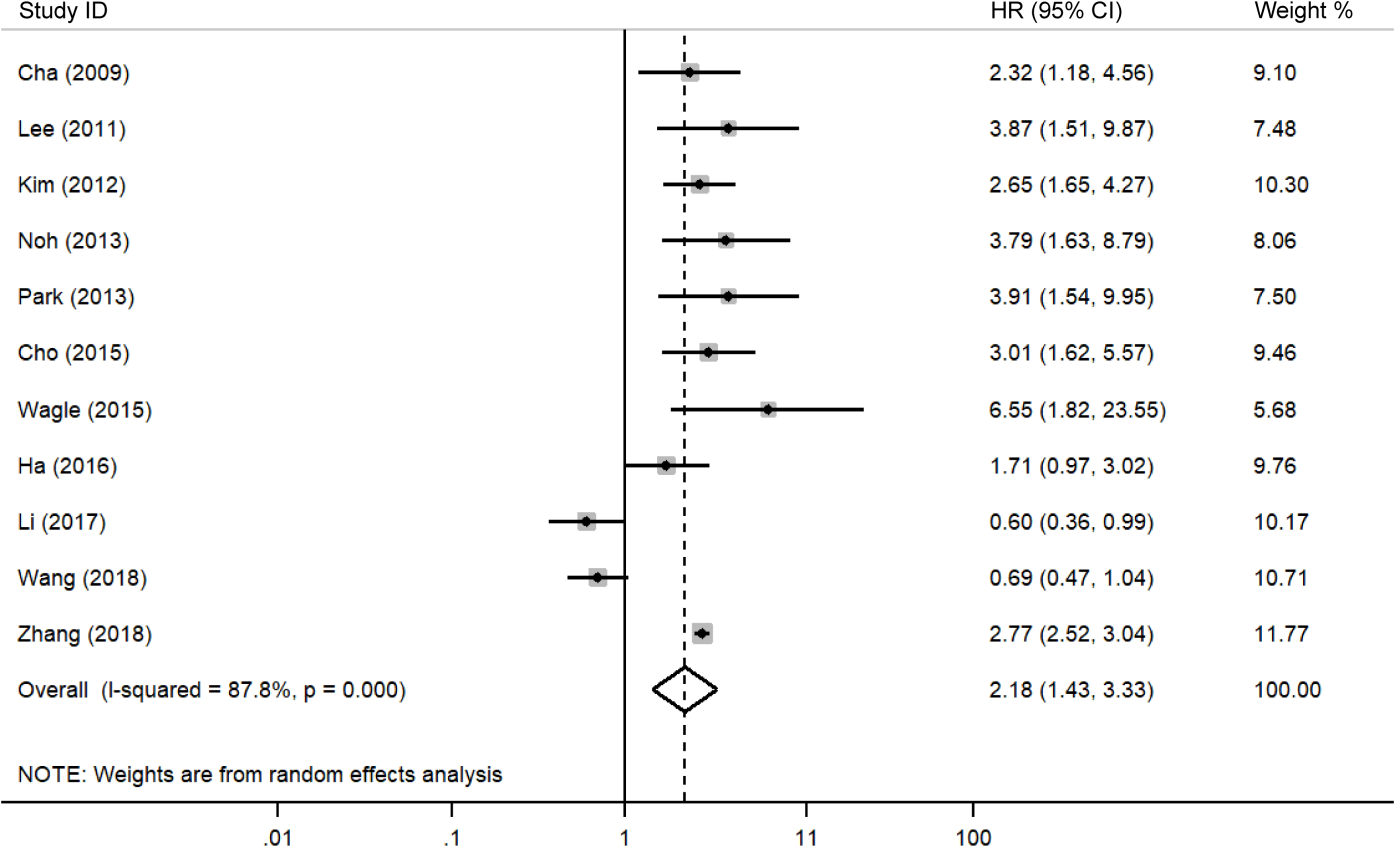
Forest plot of DBC1 expression and RFS in various cancers.

A meta-analysis was performed to evaluate the association between DBC1 expression and cancer prognosis, **Table 3** summarizes the impact of DBC1 overexpression on patient survival. **Figure 2** illustrates its relationship with OS, the analysis revealed that elevated DBC1 expression was significantly associated with poorer OS outcomes (n = 17, HR = 1.948, 95%CI: [1.280-2.964], P = 0.002, *I^2^* = 88.6).16 multivariable analysis data confirmed that patients with DBC1 overexpression exhibited significantly reduced OS (n = 16, HR = 2.014, 95%CI: [1.285-3.156], P = 0.002, *I^2^* = 89.3), a finding consistent with the results of univariable analysis (n = 12, HR = 3.154, 95%CI: [2.228-4.465], P = 0.000, *I^2^* = 57.9). Similarly, **Figure 3** presents the correlation between DBC1 expression and RFS, demonstrating that high DBC1 expression is linked to worse RFS outcomes (n = 11, HR = 2.182, 95%CI: [1.430-3.330], P = 0.000, *I^2^* = 87.8). Both univariable (n = 9, HR = 3.006, 95%CI: [2.439-3.705], P = 0.000, *I^2^* = 0.0) and multivariable (n = 10, HR = 2.123, 95%CI: [1.336-3.373], P = 0.001, *I^2^* = 89.0) analyses supported this conclusion.

**Table 3:** Survival effects of DBC1 overexpression and the prognosis of patients.

Categories	Studies (patients)	HR (95% CI)	P	I^2^ (%)	Z	P_h_	Model	P-value of Egger’ s test, Begg’ s test
OS								
Over all	17 (2841)	1.948 (1.280-2.964)	0.002	88.6	3.11	0.000	Random	0.455, 0.044
Univariable analysis	12 (1861)	3.154 (2.228-4.465)	0.000	57.9	6.47	0.006	Random	0.030, 0.005
Multivariable analysis	16 (2492)	2.014 (1.285-3.156)	0.002	89.3	3.05	0.000	Random	0.460, 0.043
RFS								
Over all	11 (1496)	2.182 (1.430-3.330)	0.000	87.8	3.62	0.000	Random	0.437, 0.119
Univariable analysis	9 (1222)	3.006 (2.439-3.705)	0.000	0.0	10.32	0.521	Fixed	0.047, 0.009
Multivariable analysis	10 (1392)	2.123 (1.336-3.373)	0.001	89.0	3.19	0.000	Random	0.410, 0.210

HR, hazard ratio; CI, confidence interval; P, P-value for statistical significance based on Z test; P_h_, P-value for heterogeneity based on Q test; OS, overall survival; RFS, recurrence-free survival.

### 3.3 Correlation between DBC1 expression and prognosis of cancer type

Subgroup analysis of DBC1 expression and OS correlation is shown in **Table 4**. Analysis by cancer type revealed that DBC1 overexpression was significantly associated with poorer OS in patients with sarcoma (n = 2, HR = 7.186, 95%CI: [2.961-17.441], P = 0.000, *I^2^* = 0.0). In contrast, no significant correlation was observed in cancers of the digestive system (n = 10, HR = 1.176, 95%CI: [0.760-1.820], P = 0.466, *I^2^* = 86.5) or reproductive system (n = 2, HR = 4.540, 95%CI: [0.868-23.741], P = 0.073, *I^2^* = 61.8). Further analysis showed that in patients with CC, DBC1 overexpression was linked to worse OS outcomes (n = 3, HR = 1.688, 95%CI: [1.352-2.107], P = 0.000, *I^2^* = 27.4). However, in GC (n = 3, HR = 1.015, 95%CI: [0.454-2.268], P = 0.971, *I^2^* = 87.8) and HCC (n = 2, HR = 0.700 95%CI: [0.295-1.664], P = 0.420, *I^2^* = 73.0), DBC1 expression status was not significantly associated with OS.

**Table 4:** Subgroup meta-analysis of the association between DBC1 expression and OS.

Categories	Studies (patients)	HR (95% CI) ^F^	P-value ^F^	HR (95% CI) ^R^	P-value ^R^	I^2^ (%)	P_h_
Country							
Korea	12 (2112)	1.529 (1.241-1.884)	0.000	2.373 (1.345-4.187)	0.003	83.9	0.000
China	5 (729)	1.666 (1.421-1.952)	0.000	1.420 (0.682-2.953)	0.349	94.4	0.000
Size							
< 100	2 (108)	3.638 (2.762-4.793)	0.000	4.038 (2.156-7.563)	0.000	25.0	0.248
100-200	12 (1731)	1.717 (1.443-2.044)	0.000	2.175 (1.336-3.540)	0.002	81.9	0.000
> 200	3 (1002)	0.748 (0.585-0.957)	0.021	0.793 (0.496-1.268)	0.333	71.1	0.031
Cancer type							
Digestive system cancer	10 (2102)	1.172 (1.010-1.359)	0.037	1.176 (0.760-1.820)	0.466	86.5	0.000
Reproductive system cancer	2 (226)	3.013 (1.492-6.082)	0.002	4.540 (0.868-23.741)	0.073	61.8	0.106
Sarcoma	2 (139)	7.186 (2.961-17.441)	0.000	7.186 (2.961-17.441)	0.000	0.0	0.764
Other	3 (374)	3.621 (2.774-4.726)	0.000	4.179 (2.079-8.401)	0.000	48.4	0.144
Cancer							
GC	3 (830)	0.768 (0.592-0.997)	0.047	1.015 (0.454-2.268)	0.971	87.8	0.000
CC	3 (685)	1.688 (1.352-2.107)	0.000	1.720 (1.249-2.369)	0.001	27.4	0.252
HCC	2 (318)	0.669 (0.429-1.045)	0.077	0.700 (0.295-1.664)	0.420	73.0	0.054
Cutoff value							
Staining cells ≥30	6 (1400)	1.180 (0.907-1.536)	0.218	1.777 (0.884-3.572)	0.107	82.5	0.000
SI × PP ≥7	4 (620)	1.604 (1.304-1.973)	0.000	1.603 (0.768-3.348)	0.209	87.8	0.000
SI + PP ≥6	3 (243)	3.814 (2.151-6.764)	0.000	4.357 (1.937-9.800)	0.000	42.1	0.178
Other	4 (578)	1.772 (1.431-2.193)	0.000	1.783 (0.503-6.321)	0.371	95.6	0.000

HR ^F^, hazard ratio were derived from fixed-effect model; HR ^R^, hazard ratio were derived from random-effect model; CI, confidence interval; P-value ^F^, P-value were derived from fixed-effect model; P-value ^R^, P-value were derived from random-effect model; P_h_, P-value for heterogeneity based on Q test; GC, gastric cancer; CC, Colorectal Cancer; HCC, hepatocellular carcinoma; SI, staining intensity; PP, percentage of positive.

**Table 5** presents the results of a subgroup analysis examining the association between DBC1 expression and RFS in cancer patients. Among individuals diagnosed with reproductive system cancers (n = 2, HR = 3.244, 95%CI: [1.938-5.427], P = 0.000, *I^2^* = 0.0) and sarcomas (n = 1, HR = 6.555, 95%CI: [1.825-23.546], P = 0.004), elevated DBC1 expression was identified as a predictor of poorer RFS. Conversely, no significant relationship was observed between DBC1 expression and RFS in patients with digestive system cancers (n = 5, HR = 1.322, 95%CI: [0.704-2.480], P = 0.385, *I^2^* = 86.9). Further analysis of specific cancer types revealed no significant association in GC (n = 2, HR = 1.229, 95%CI: [0.377-4.007], P = 0.732, *I^2^* = 89.0) or HCC (n = 2, HR = 1.004, 95%CI: [0.361-2.798], P = 0.993, *I^2^* = 86.4).

**Table 5:** Subgroup meta-analysis of the association between DBC1 expression and RFS.

Categories	Studies (patients)	HR (95% CI) ^F^	P-value ^F^	HR (95% CI) ^R^	P-value ^R^	I^2^ (%)	P_h_
Country							
Korea	8 (1103)	2.752 (2.154-3.516)	0.000	2.752 (2.154-3.516)	0.000	0.0	0.492
China	3 (393)	2.448 (2.235-2.681)	0.000	1.067 (0.339-3.352)	0.912	97.3	0.000
Size							
< 100	2 (108)	2.779 (2.528-3.055)	0.000	3.332 (1.662-6.680)	0.001	42.5	0.187
100-200	8 (1187)	1.976 (1.581-2.470)	0.000	2.280 (1.392-3.735)	0.001	78.1	0.000
> 200	1 (201)	0.694 (0.465-1.035)	0.073	0.694 (0.465-1.035)	0.073	–	–
Cancer type							
Digestive system cancer	5 (861)	1.191 (0.952-1.490)	0.126	1.322 (0.704-2.480)	0.385	86.9	0.000
Reproductive system cancer	2 (226)	3.244 (1.938-5.427)	0.000	3.244 (1.938-5.427)	0.000	0.0	0.661
Sarcoma	1 (35)	6.555 (1.825-23.546)	0.004	6.555 (1.825-23.546)	0.004	–	–
Other	3 (374)	2.787 (2.537-3.061)	0.000	2.787 (2.537-3.061)	0.000	0.0	0.594
Cancer							
GC	2 (378)	0.950 (0.673-1.340)	0.769	1.229 (0.377-4.007)	0.732	89.0	0.003
HCC	2 (318)	0.948 (0.651-1.379)	0.778	1.004 (0.361-2.798)	0.993	86.4	0.007
Cutoff value							
Staining cells ≥30	4 (599)	2.393 (1.664-3.441)	0.000	2.439 (1.646-3.614)	0.000	12.2	0.332
SI × PP ≥7	2 (284)	1.308 (0.927-1.846)	0.126	1.266 (0.295-5.427)	0.751	94.4	0.000
SI + PP ≥6	2 (139)	3.482 (1.999-6.066)	0.000	3.600 (1.891-6.851)	0.000	13.6	0.282
Other	3 (474)	2.581 (2.354-2.829)	0.000	1.883 (0.672-5.276)	0.229	95.5	0.000

HR ^F^, hazard ratio were derived from fixed-effect model; HR ^R^, hazard ratio were derived from random-effect model; CI, confidence interval; P-value ^F^, P-value were derived from fixed-effect model; P-value ^R^, P-value were derived from random-effect model; P_h_, P-value for heterogeneity based on Q test; GC, gastric cancer; HCC, hepatocellular carcinoma; SI, staining intensity; PP, percentage of positive.

### 3.4 Correlation between DBC1 expression and prognosis in different countries and sample sizes

Subgroup analysis indicated that elevated DBC1 expression was associated with reduced OS (n = 12, HR = 2.373, 95%CI: [1.345-4.187], P = 0.003, *I^2^* = 83.9) and RFS (n = 8, HR = 2.752, 95%CI: [2.154-3.516], P = 0.000, *I^2^* = 0.0) in Korean patients. In contrast, no significant relationship was observed between DBC1 expression and OS (n = 5, HR = 1.420, 95%CI: [0.682-2.953], P = 0.349, *I^2^* = 94.4) or RFS (n = 3, HR = 1.067, 95%CI: [0.339-3.352], P = 0.912, *I^2^* = 97.3) in Chinese patients.

Further analysis stratified by study sample size revealed that studies with small (< 100, n = 2, HR = 3.638, 95%CI: [2.762-4.793], P = 0.000, *I^2^* = 25.0) and medium (100–200, n = 12, HR = 2.175, 95%CI: [1.336-3.540], P = 0.002, *I^2^* = 81.9) sample sizes indicated a negative correlation between DBC1 overexpression and OS. However, no statistically significant differences were observed in studies with large sample sizes (>200, n = 3, HR = 0.793, 95%CI: [0.496-1.268], P = 0.333, *I^2^* = 71.1). Similarly, the association between elevated DBC1 expression and worse RFS was evident only in studies with small (n = 2, HR = 2.779, 95%CI: [2.528-3.055], P = 0.000, *I^2^* = 42.5) and medium (n = 8, HR = 2.280, 95%CI: [1.392-3.735], P = 0.001, *I^2^* = 78.1) sample sizes.

### 3.5 Correlation between different cutoff values and prognosis

The use of varying cutoff criteria to define DBC1 positivity resulted in inconsistent findings in the subgroup analysis. Studies employing a cutoff of SI + PP ≥6 demonstrated that DBC1 overexpression was associated with reduced OS (n = 3, HR = 3.814, 95%CI: [2.151-6.764], P = 0.000, *I^2^* = 42.1) and RFS (n = 2, HR = 3.482, 95%CI: [1.999-6.066], P = 0.000, *I^2^* = 13.6). Studies defining DBC1 positivity as staining cells ≥30% identified a correlation between elevated DBC1 expression and poorer RFS (n = 4, HR = 2.393, 95%CI: [1.664-3.441], P = 0.000, *I^2^* = 12.2).

### 3.6 Correlation of DBC1 expression with clinicopathologic characters

**Figure 4:**
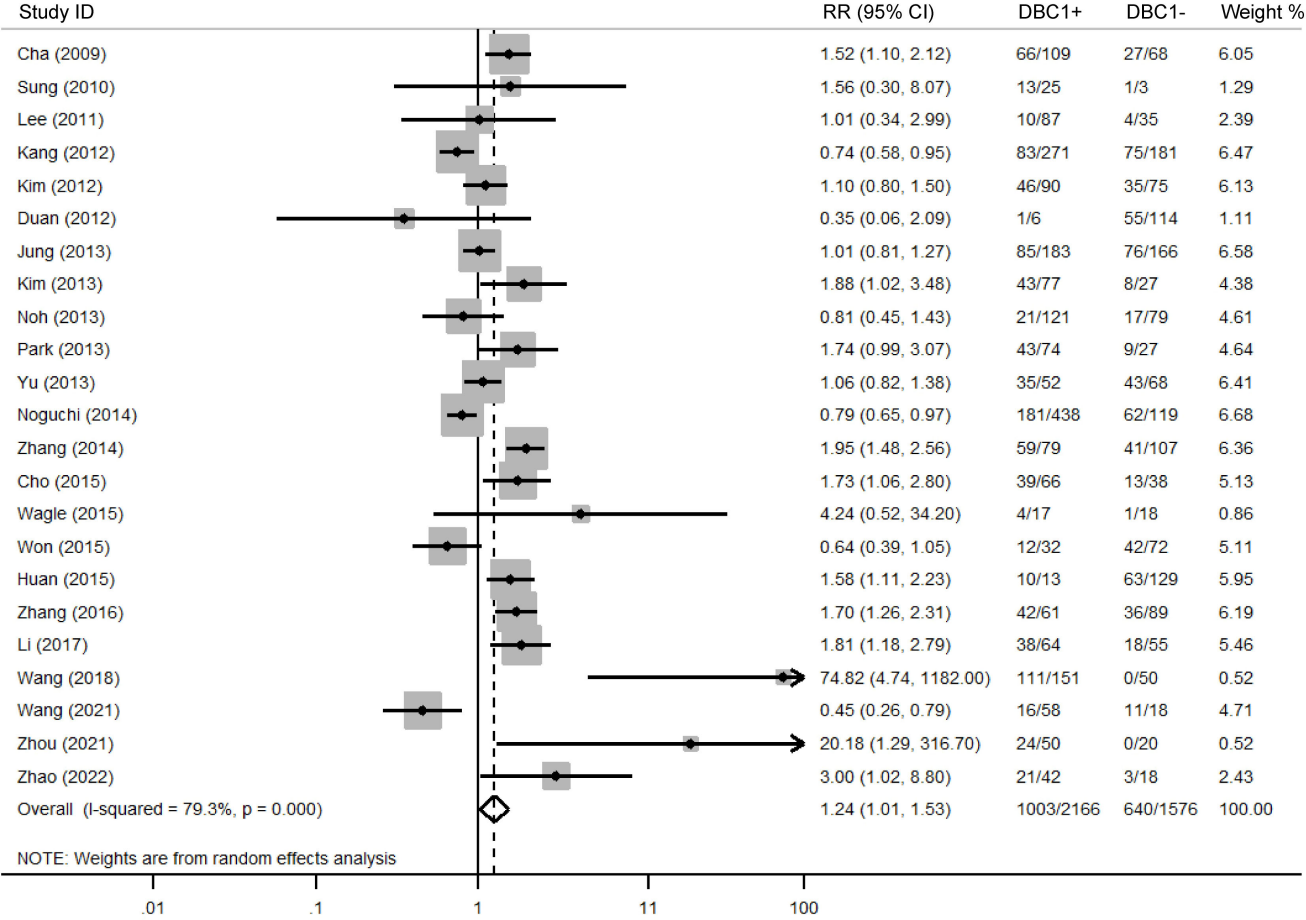
Forest plot of DBC1 expression and TNM stage in various cancers.

**Figure 5:**
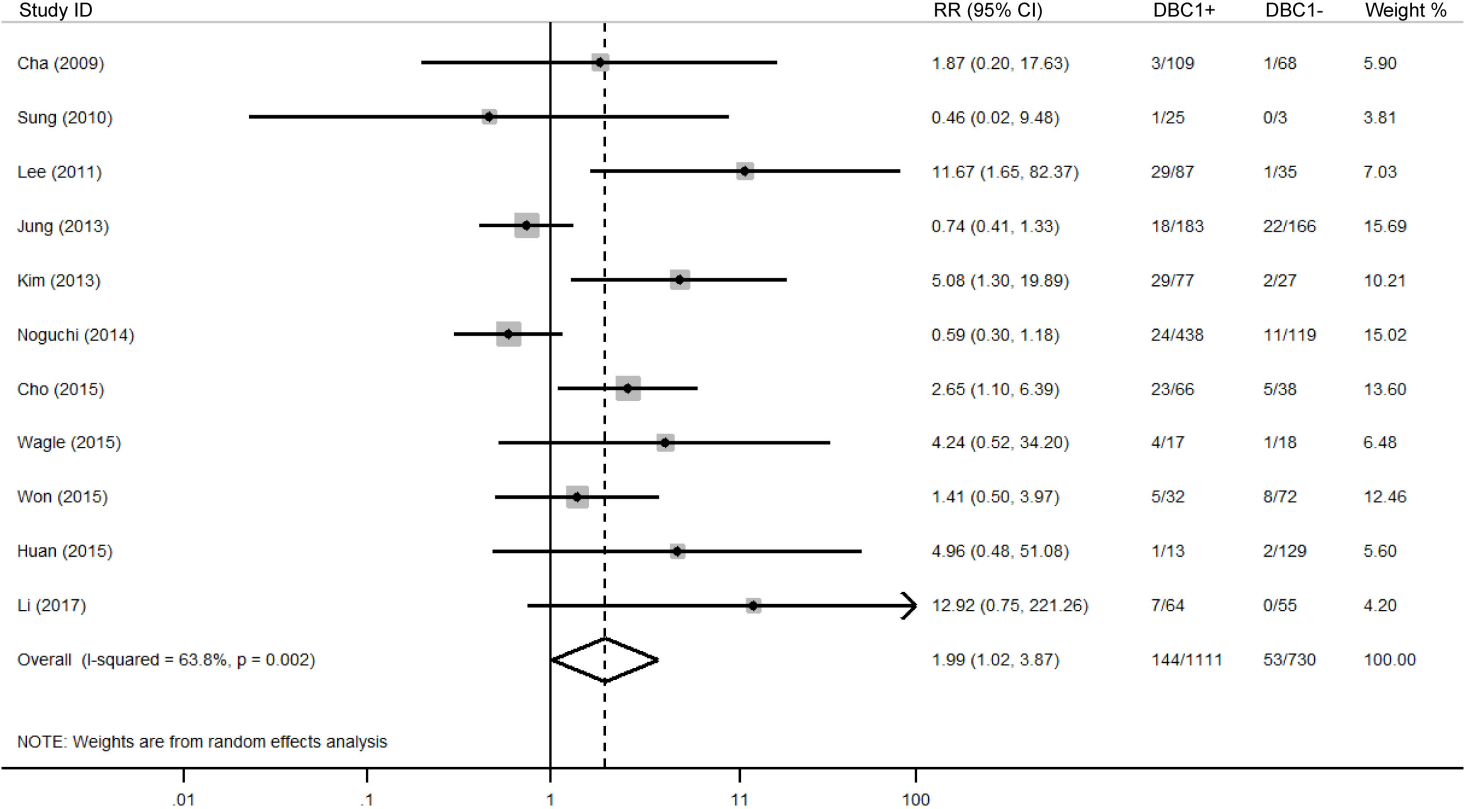
Forest plot of DBC1 expression and distant metastasis in various cancers.

**Figure 6:**
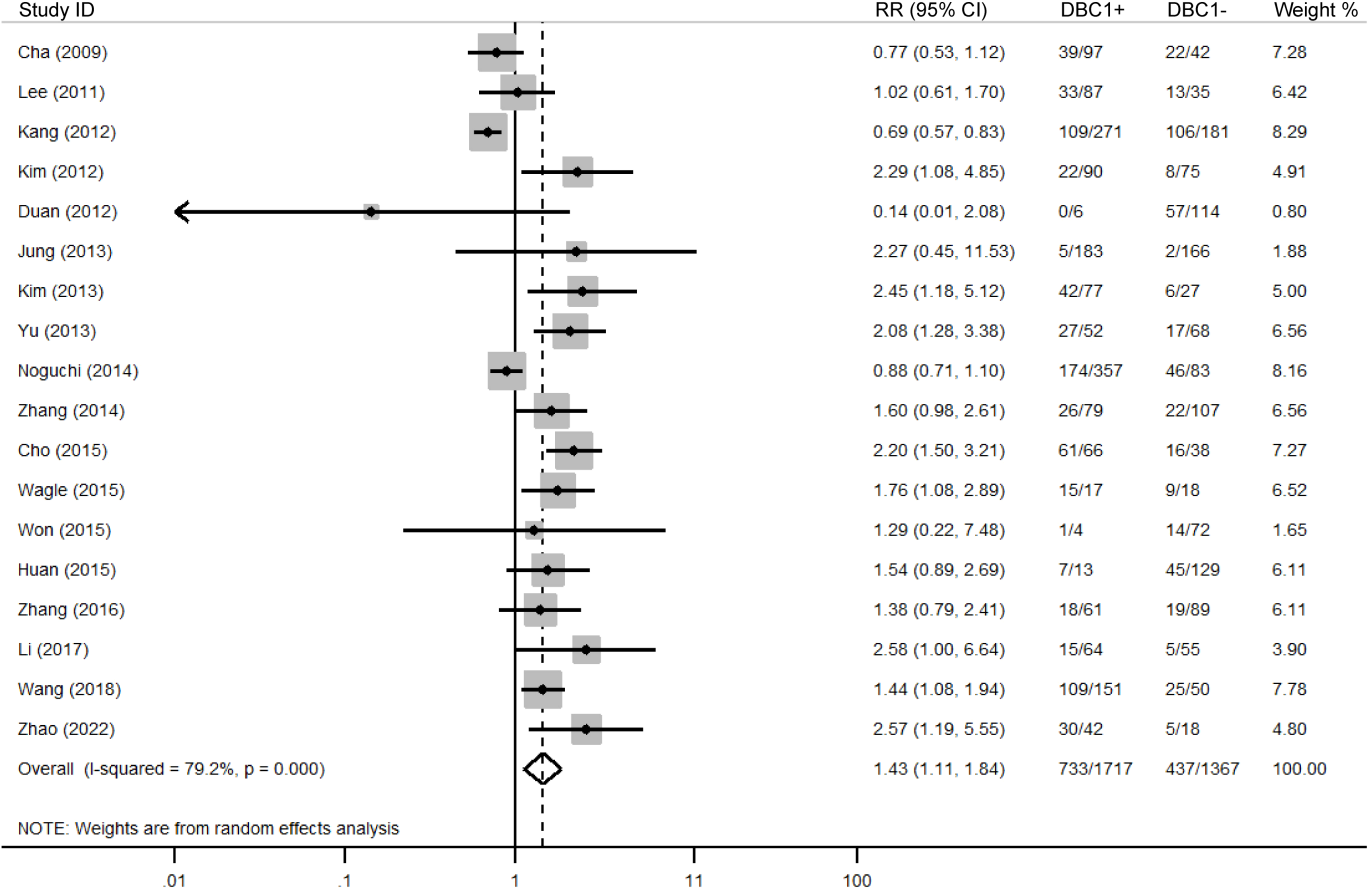
Forest plot of DBC1 expression and histologic grade in various cancers.

The association between DBC1 expression and various clinicopathological features was analyzed, with the findings summarized in **Table 6**. High DBC1 expression was significantly correlated with advanced TNM stage (n = 23, RR = 1.245, 95%CI: [1.012-1.531], P = 0.038, *I^2^* = 79.3, **Figure 4**), increased incidence of distant metastasis (n = 11, RR = 1.987, 95%CI: [1.021-3.866], P = 0.043, *I^2^* = 63.8, **Figure 5**), and poorer histologic grade (n = 18, RR = 1.433, 95%CI: [1.115-1.843], P = 0.005, *I^2^* = 79.2, **Figure 6**). However, no significant association was observed between DBC1 expression and LN metastasis, tumor size, tumor invasion, vascular invasion, age, gender, or P53 expression (**Supplementary Figure S1**).

### 3.7 Correlation between DBC1 expression and clinicopathologic characters of cancer type

Subgroup analysis of the correlation between DBC1 expression and clinicopathological features is summarized in **Supplementary Table S2**. The findings indicated that patients with reproductive system cancers and DBC1 overexpression were more likely to present with advanced TNM stage (n = 4, RR = 1.760, 95%CI: [1.180-2.627], P = 0.006, *I^2^* = 0.0), distant metastasis (n = 3, RR = 3.918, 95%CI: [1.801-8.522], P = 0.001, *I^2^* = 48.5), and poor histologic grade (n = 3, RR = 1.751, 95%CI: [1.000-3.065], P = 0.050, *I^2^* = 69.9). Likewise, sarcoma patients with elevated DBC1 expression exhibited significant associations with advanced TNM stage (n = 2, RR = 2.063, 95%CI: [1.146-3.715], P = 0.016, *I^2^* = 0.0), increased incidence of distant metastasis (n = 2, RR = 4.875, 95%CI: [1.537-15.458], P = 0.007, *I^2^* = 0.0), and unfavorable histologic grade ((n = 2, RR = 2.112, 95%CI: [1.337-3.338], P = 0.001, *I^2^* = 0.0). In reproductive system cancers, the relationship between DBC1 overexpression and tumor size yielded inconsistent results, a meta-analysis of two studies suggested that DBC1 overexpression was associated with smaller tumor size (n = 2, RR = 0.616, 95%CI: [0.409-0.928], P = 0.020, *I^2^* = 0.0).

For specific cancer types, CC patients with elevated DBC1 expression exhibited a trend toward poorer histologic grade (n = 3, RR = 1.546, 95%CI: [1.080-2.214], P = 0.017, *I^2^* = 0.0), while no statistically significant differences were observed in other cases.

### 3.8 Correlation between DBC1 expression and clinicopathologic characters in different countries and sample sizes

Subgroup analysis of the relationship between DBC1 expression and clinicopathological characteristics was conducted across cancer patients from different countries. Among Chinese patients, DBC1 overexpression was significantly associated with adverse prognostic factors, including advanced TNM stage (n = 10, RR = 1.522, 95%CI: [1.005-2.303], P = 0.047, *I^2^* = 84.8), LN metastasis (n = 7, RR = 1.527, 95%CI: [1.110-2.103], P = 0.009, *I^2^* = 74.3), distant metastasis (n = 2, RR = 9.696, 95%CI: [1.231-76.363], P = 0.031, *I^2^* = 0.0), worse histologic grade (n = 8, RR = 1.598, 95%CI: [1.323-1.930], P = 0.000, *I^2^* = 5.7), increased tumor invasion (n = 2, RR = 2.661, 95%CI: [1.489-4.756], P = 0.001, *I^2^* = 0.0) and vascular invasion (n = 1, RR = 2.460, 95%CI: [1.196-5.060], P = 0.014). In Korean patients, DBC1 overexpression was linked to a higher risk of distant metastasis (n = 8, RR = 2.110, 95%CI: [1.004-4.435], P = 0.049, *I^2^* = 59.9) and vascular invasion (n = 4, RR = 1.833, 95%CI: [1.241-2.705], P = 0.002, *I^2^* = 38.7).

An additional subgroup analysis stratified by sample size showed studies with moderate sample sizes (100–200) demonstrated significant correlations between DBC1 overexpression and unfavorable outcomes, including advanced TNM stage (n = 14, RR = 1.366, 95%CI: [1.135-1.645], P = 0.001, *I^2^* = 61.9), LN metastasis (n = 12, RR = 1.396, 95%CI: [1.137-1.714], P = 0.001, *I^2^* = 59.9), distant metastasis (n = 7, RR = 3.737, 95%CI: [2.185-6.393], P = 0.000, *I^2^* = 9.8), histologic grade (n = 12, RR = 1.550, 95%CI: [1.172-2.050], P = 0.002, *I^2^* = 59.8), and tumor invasion (n = 2, RR = 1.317, 95%CI: [1.105-1.571], P = 0.002, *I^2^* = 0.0). In studies with large sample sizes (>200), a statistically significant association was observed between DBC1 expression and tumor size (n = 1, RR = 2.907, 95%CI: [1.578-5.354], P = 0.001). By contrast, studies with small sample sizes (<100) suggested that elevated DBC1 expression was correlated with poorer histologic grade (n = 2, RR = 2.123, 95%CI: [1.352-3.336], P = 0.001, *I^2^* = 0.0) and increased tumor invasion (n = 2, RR = 2.661, 95%CI: [1.489-4.756], P = 0.001, *I^2^* = 0.0).

### 3.9 Correlation between different cutoff values and clinicopathologic characters

Subgroup analyses demonstrated that studies using a cutoff of staining cells ≥30% to define DBC1 positivity identified an association between DBC1 overexpression and a more favorable histologic grade (n = 4, RR = 0.754, 95%CI: [0.642-0.885], P = 0.001, *I^2^* = 26.3). Conversely, studies employing SI × PP ≥ 7 as the criterion for DBC1 positivity identified significant correlations between elevated DBC1 expression and adverse outcomes, including advanced TNM stage (n = 7, RR = 1.446, 95%CI: [1.146-1.824], P = 0.002, *I^2^* = 66.1), LN metastasis (n = 6, RR = 1.523, 95%CI: [1.190-1.950], P = 0.001, *I^2^* = 68.8), distant metastasis (n = 2, RR = 9.696, 95%CI: [1.231-76.363], P = 0.031, *I^2^* = 0.0), and poorer histologic grade (n = 7, RR = 1.663, 95%CI: [1.307-2.116], P = 0.000, *I^2^* = 1.2). Similarly, studies using SI + PP ≥ 6 as the cutoff revealed that DBC1 overexpression was significantly associated with advanced TNM stage (n = 3, RR = 1.874, 95%CI: [1.287-2.729], P = 0.001, *I^2^* = 0.0), increased distant metastasis (n = 3, RR = 3.500, 95%CI: [1.732-7.076], P = 0.000, *I^2^* = 0.0), and poorer histologic grade (n = 3, RR = 2.157, 95%CI: [1.608-2.893], P = 0.000, *I^2^* = 0.0).

### 3.10 Meta-regression

To explore the potential sources of heterogeneity, meta-regression analyses were performed using covariates including cancer type, cancer, country, sample size, and cutoff value (**Supplementary Table S3**). In the meta-regression for OS, residual heterogeneity was reduced to 74.72%, with the model accounting for 58.85% of the between-study variance. Among the covariates, cancer (β = −0.13, P = 0.044) and sample size (β = −1.10, P = 0.038) emerged as significant contributors to heterogeneity, while the remaining variables did not show statistically significant effects.

For RFS, the meta-regression fully explained the heterogeneity, with the residual I² reduced to 0% and the model accounting for 100.00% of the variance. Among the examined factors, only country was identified as a significant source of heterogeneity (β = −0.99, P = 0.013), indicating that patients from Korea with high DBC1 expression exhibited significantly worse RFS compared to those from China.

### 3.11 Sensitivity analysis

**Figure 7:**
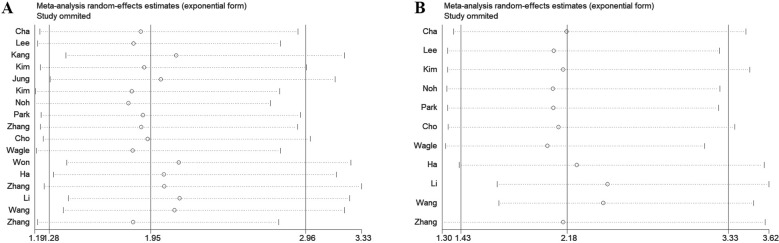
Sensitive analysis of OS **(A)** and RFS **(B)** for patients in various cancers.

To assess the robustness of the meta-analysis results, a leave-one-out sensitivity analysis was performed by sequentially excluding each study to evaluate its impact on the overall findings. The analysis revealed that in studies examining the association between DBC1 expression and OS or RFS, the exclusion of any single study did not affect the final outcomes (**Figure 7**). Similarly, sensitivity analysis of studies investigating the relationship between DBC1 expression and clinicopathological characteristics yielded consistent results (**Supplementary Figure S2**).

### 3.12 Publication bias

**Figure 8:**
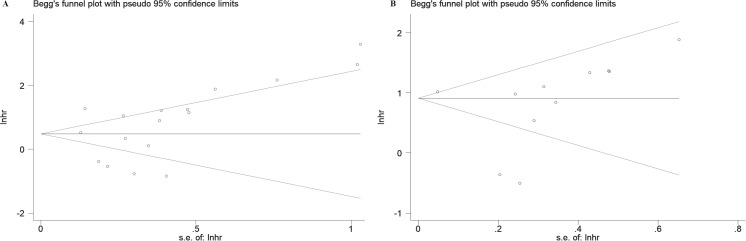
Begg' s funnel plots of publication bias for survival in various cancers. **(A)** OS. **(B)** RFS.

To assess publication bias, Egger’s test, Begg’s test, and funnel plot analysis were conducted. The funnel plots indicated no significant asymmetry in the included studies concerning prognostic indicators (**Figure 8**) or pathological features (**Supplementary Figure S3**). Similarly, the results of Egger’s and Begg’s tests showed no significant publication bias in the overall analyses of OS and RFS (**Table 3**). However, Egger’s test identified potential publication bias in studies examining distant metastasis, histologic grade, and vascular invasion among pathological characteristics (**Table 6**).

**Table 6:** The associations of DBC1 overexpression with the clinicopathological characteristics of the study patients.

Clinicopathological parameters	Studies (patients)	RR (95% CI) ^F^	P-value ^F^	RR (95% CI) ^R^	P-value ^R^	I^2^ (%)	P_h_	P-value of Egger’ s test, Begg’ s test
TNM stage	23 (3742)	1.243 (1.144-1.351)	0.000	**1.245 (1.012-1.531)**	**0.038**	79.3	0.000	0.122, 0.316
LN metastasis	18 (3209)	1.142 (1.047-1.246)	0.003	1.193 (0.967-1.473)	0.100	77.8	0.000	0.807, 0.762
Distant metastasis	11 (1841)	1.640 (1.198-2.244)	0.002	**1.987 (1.021-3.866)**	**0.043**	63.8	0.002	0.022, 0.533
Tumor size	12 (1449)	1.104 (0.961-1.267)	0.163	1.047 (0.815-1.345)	0.721	67.8	0.000	0.853, 0.945
Histologic grade	18 (3084)	1.170 (1.060-1.292)	0.002	**1.433 (1.115-1.843)**	**0.005**	79.2	0.000	0.014, 0.762
Tumor invasion	6 (1320)	1.068 (0.975-1.171)	0.155	1.196 (0.929-1.541)	0.165	80.7	0.000	0.160, 0.452
Vascular invasion	6 (1266)	1.172 (0.984-1.397)	0.076	1.595 (0.926-2.749)	0.093	77.2	0.001	0.028, 0.707
Age	24 (3941)	1.122 (1.048-1.202)	0.001	1.080 (0.972-1.200)	0.151	51.2	0.002	0.783, 0.673
Gender	20 (3627)	1.027 (0.979-1.076)	0.273	1.011 (0.968-1.055)	0.629	9.9	0.332	0.333, 0.538
P53	9 (1622)	1.264 (1.100-1.452)	0.001	1.373 (0.928-2.031)	0.113	81.6	0.000	0.256, 0.466

RR ^F^, relative risk were derived from fixed-effect model; RR ^R^, relative risk were derived from random-effect model; CI, confidence interval; P-value ^F^, P-value were derived from fixed-effect model; P-value ^R^, P-value were derived from random-effect model; P^h^, P-value for heterogeneity based on Q test; LN, lymph node.Bold indicates statistically significant differences.

## 4 Discussion

A major focus in oncology research is the identification of biomarkers capable of reliably predicting cancer prognosis. The absence of DBC1 expression in breast cancer patients positions it as a promising candidate for further investigation. Despite a growing body of research exploring the role of DBC1 across various cancer types, the results remain inconclusive, leading to ambiguity regarding the prognostic implications of DBC1 expression in cancer patients (6, 42). In response to this, a comprehensive meta-analysis was conducted using data from five authoritative databases: PubMed, Web of Science, Embase, CNKI, and Wanfang. The aim was to synthesize existing evidence on the prognostic value of DBC1 in cancer patients. Our meta-analysis, incorporating data from 25 studies involving 4014 patients, demonstrated that DBC1 expression is a significant predictor of both OS (n = 17, HR = 1.948, 95% CI: [1.280-2.964], P = 0.002, *I^2^* = 88.6) and RFS (n = 11, HR = 2.182, 95% CI: [1.430-3.330], P < 0.001, *I^2^* = 87.8). Furthermore, this study marks the first systematic assessment of the association between DBC1 expression and clinicopathological characteristics in diverse cancer cohorts. Our findings indicate that DBC1 overexpression is correlated with a more advanced TNM stage (n = 23, RR = 1.245, 95% CI: [1.012-1.531], P = 0.038, *I^2^* = 79.3), a higher incidence of distant metastasis (n = 11, RR = 1.987, 95% CI: [1.021-3.866], P = 0.043, *I^2^* = 63.8), and a more aggressive histologic grade (n = 18, RR = 1.433, 95% CI: [1.115-1.843], P = 0.005, *I^2^* = 79.2). We enhanced the relevance of our findings through subgroup analyses, which separately evaluated the prognostic and clinicopathological predictive value of DBC1 expression across different countries, cancer types, sample sizes, cancer, and cutoff values. To further investigate the sources of heterogeneity observed in the pooled analysis, we conducted meta-regression using cancer type, specific cancer classification, study country, sample size, and cutoff value as covariates. For OS, cancer (β = −0.13, P = 0.044) and study sample size (β = −1.10, P = 0.038) emerged as significant contributors to heterogeneity, jointly explaining 58.85% of between-study variance. This suggests that DBC1’s prognostic impact varies by cancer lineage, with smaller studies potentially overestimating effect sizes. In contrast, RFS heterogeneity was primarily driven by geographical differences (β = −0.99, P = 0.013), likely reflecting regional variations in treatment protocols or genetic backgrounds. Sensitivity analysis and publication bias analyses further corroborated the reliability of our findings.

Genomic instability and mutations are key contributors to tumorigenesis. Since its discovery, the relationship between DBC1 and cancer has been marked by significant contradictions and uncertainties. Subsequent studies have confirmed that DBC1 exerts diverse effects on various pathophysiological processes. Through its structural domains, DBC1 interacts with multiple proteins, including SIRT1, HDAC3, and SUV39H1, thereby influencing processes such as immunity, aging, and metabolism (6). One of DBC1’s most critical roles is its function as an endogenous inhibitor of SIRT1, a pivotal deacetylase with numerous downstream targets. The LZ domain of DBC1 inhibits SIRT1 activity by competing with its ESA region, thereby modulating acetylation levels and affecting a variety of disease processes (43–45). DBC1 regulates cancer development and progression, particularly through its interaction with P53. It inhibits the SIRT1-mediated deacetylation of P53 and FOXO3, maintaining P53 in a hyperacetylated state and promoting P53-dependent apoptosis in cancer cells (7). Furthermore, DBC1 enhances P53 stability by interfering with MDM2- and CBP-mediated polyubiquitination, independent of SIRT1 activity (46, 47). In addition to P53, DBC1 affects cancer through other downstream pathways. While SIRT1 can promote cancer progression by enhancing c-MYC activity, DBC1 counteracts this process, thereby exhibiting anticancer effects (48). However, DBC1 also demonstrates cancer-promoting activity. For instance, TP53 mutations, present in approximately 42% of cancer patients, are associated with accelerated cancer progression, resistance to therapy, and poor prognosis (49, 50). It has been proposed that TP53 mutations, which disrupt DBC1-mediated regulation of P53-dependent apoptosis, may contribute to the contradictory role of DBC1 in cancer (6).

Our study confirms that DBC1 expression can serve as a predictor of poor prognosis to a certain extent; however, its prognostic value must still be interpreted in the context of individual patient characteristics. The biological role of DBC1 varies significantly across different cancer types, primarily due to its involvement in diverse signaling pathways and interactions with key molecular partners. Through these mechanisms, DBC1 regulates cancer cell proliferation, apoptosis, migration, and sensitivity to therapy. ERα and AR are key target proteins of DBC1, and their functions are largely influenced by acetylation levels. In breast cancer, DBC1 modulates the transcriptional activity of ERα through cooperative interaction, thereby promoting the progression of hormone-dependent breast cancer. DBC1 inhibits SIRT1-mediated deacetylation of both ERα and AR. Experimental evidence has demonstrated that increased DBC1 expression markedly diminishes the tumor-suppressive effects of SIRT1 in breast cancer, significantly shortening the survival of both ERα-positive and ERα-negative patients. This suggests the broad prognostic significance of DBC1 across different molecular subtypes of breast cancer (14, 51, 52). In CRC, DBC1 promotes the formation of the LEF1-β-catenin complex in the Wnt signaling pathway by reducing SIRT1-mediated β-catenin deacetylation, thereby enhancing Wnt-β-catenin signaling and worsening prognosis in CRC patients (53, 54). Additionally, DBC1 supports cancer cell survival through multiple mechanisms, including interaction with HSP60 and epigenetic regulation of chromatin structure and histone modifications (53–55). It facilitates enhancer-promoter interactions in cancer cells and contributes to the formation of super-enhancers, particularly in CRC, further promoting oncogenesis (56). Recent studies have further elucidated the oncogenic role of DBC1 in gastric cancer. Huan et al. (13) reported that DBC1 is upregulated in gastric cancer tissues and is significantly associated with advanced TNM stage and lymph node metastasis. High DBC1 expression serves as an independent predictor of poor prognosis. Mechanistically, DBC1 promotes anoikis resistance and metastatic potential in tumor cells by activating the IKKβ/NF-κB signaling pathway and upregulating anti-apoptotic genes such as c-FLIP and Bcl-xL. These findings highlight not only the prognostic significance of DBC1 but also its potential as a novel therapeutic target for preventing gastric cancer metastasis. In hepatocellular carcinoma, elevated DBC1 expression is linked to higher TNM stages, poorer histological differentiation, and shorter survival times, suggesting its utility as an important marker for patient stratification and therapeutic planning. Notably, DBC1 expression shows a progressive increase during the transition from precancerous lesions to malignant tumors in HCC, indicating its potential value in early cancer diagnosis (31).

Although our study confirmed that DBC1 expression is significantly associated with TNM stage, distant metastasis, and histologic grade—clearly demonstrating its value in overall prognostic assessment—we also observed no significant correlation between DBC1 expression and traditional pathological features such as LN metastasis, tumor size, tumor invasion, or vascular invasion. Several factors may account for these negative findings. First, DBC1 may be more involved in regulating systemic metastatic potential rather than local invasive behaviors. For example, it has been shown to promote the expression of MACC1, a gene implicated in distant metastasis. Second, hypoxic conditions within the tumor microenvironment may lead to the ubiquitin-mediated degradation of DBC1 via SIAH2, thereby affecting its stability and expression in different lesions (57). Additionally, the function of DBC1 may vary across cancer types depending on its interactions with specific signaling pathways and proteins. In certain tissues, DBC1 may be upregulated but fail to sufficiently activate oncogenic pathways, resulting in no apparent association with pathological indicators. In summary, DBC1 contributes to cancer progression through diverse molecular mechanisms and holds promise as a prognostic biomarker. However, its clinical applicability may vary across tumor types. Future studies should incorporate cancer-type-specific analyses, protein interaction networks, and molecular subtyping to further clarify the scope and limitations of DBC1 as a tumor marker.

This study has several limitations that must be acknowledged. First, a substantial degree of heterogeneity was observed in the results, while subgroup analyses accounted for some variability, inter-study heterogeneity persisted. This heterogeneity primarily stemmed from methodological differences in assessing DBC1 expression and varying criteria used to define DBC1 positivity. Subgroup analyses based on quantifiable cutoff values partially mitigated this issue by reducing heterogeneity within groups. However, these subgroups were broadly defined. For instance, studies utilizing cutoff values of 30% and 70% for DBC1 positivity were both categorized under the “Staining cells ≥ 30%” subgroup, and studies with cutoff values of SI + PP ≥ 6 and SI + PP ≥ 7 were grouped into the “SI + PP ≥ 6” category. Consequently, variability within subgroups contributed to intergroup differences. Given the wide range of cutoff values, we were unable to identify a more optimal solution. Second, DBC1 exerts distinct mechanisms across different cancer types, necessitating individualized analyses to elucidate its role in each cancer. Due to the limited number of studies, subgroup analyses were restricted to cancers with sufficient data, such as GC, CC, and HCC. This constraint likely contributed to unresolved heterogeneity. Third, the quality of the included studies varied, as most were retrospective in design, inherently limiting the overall quality of evidence. Future studies should aim to conduct large-scale, prospective, and multicenter investigations to validate the prognostic value of DBC1 across diverse populations and tumor types. In addition, mechanistic studies integrating molecular subtyping and individual patient data may help to clarify DBC1’s dual role and facilitate its clinical application as a biomarker. Fourth, the study population comprised patients exclusively from China, Korea, and Japan, all of Asian ethnicity. The absence of data from other ethnicities limits the generalizability of these findings. Fifth, potential publication bias was identified in the analysis of DBC1 expression and its associations with distant metastasis, histologic grade, and vascular invasion. This bias may arise from a preference for publishing positive results or stringent inclusion criteria applied in the studies. Finally, this study was limited to publications written in English or Chinese and did not include grey literature, such as unpublished studies or conference abstracts. Consequently, the presence of publication bias cannot be entirely excluded, which may affect the comprehensiveness and generalizability of our findings.

## 5 Conclusion

In summary, this study demonstrates that DBC1 overexpression is significantly associated with reduced OS and RFS in cancer patients. Regarding pathological characteristics, DBC1 overexpression was identified as a predictor of advanced TNM stage, distant metastasis, and poorer histologic grade. However, no significant associations were observed with LN metastasis, tumor size, tumor invasion, or vascular invasion. These findings highlight the potential of DBC1 as a novel prognostic biomarker and a predictor of specific pathological features in cancer. Nonetheless, the study’s limitations underscore the need for further prospective research to comprehensively elucidate the relationship between DBC1 expression and prognostic or clinicopathological parameters in cancer patients. Future studies should aim to reduce inter-sample variability and adopt standardized methodologies to enhance the robustness and generalizability of findings.

## Data Availability

The original contributions presented in the study are included in the article/**Supplementary Material**. Further inquiries can be directed to the corresponding authors.

## Supplementary material

Supplementary Figure 1 Forest plot of DBC1 expression and other clinicopathological characteristics in various cancers.

Supplementary Figure 2 Sensitive analysis of clinicopathological characteristics for patients in various cancers.

Supplementary Figure 3 Begg’s funnel plots of publication bias for clinicopathological characteristics in various cancers.

## References

[1] Sung H Ferlay J Siegel RL Laversanne M Soerjomataram I Jemal A . Global cancer statistics 2020: globocan estimates of incidence and mortality worldwide for 36 cancers in 185 countries. CA Cancer J Clin. (2021) 71:209–49. doi: 10.3322/caac.21660, PMID: 33538338

[2] Siegel RL Miller KD Fuchs HE Jemal A . Cancer statistics, 2022. CA Cancer J Clin. (2022) 72:7–33. doi: 10.3322/caac.21708, PMID: 35020204

[3] Miller KD Nogueira L Devasia T Mariotto AB Yabroff KR Jemal A . Cancer treatment and survivorship statistics, 2022. CA Cancer J Clin. (2022) 72:409–36. doi: 10.3322/caac.21731, PMID: 35736631

[4] Hamaguchi M Meth JL von Klitzing C Wei W Esposito D Rodgers L . Dbc2, a candidate for a tumor suppressor gene involved in breast cancer. Proc Natl Acad Sci U.S.A. (2002) 99:13647–52. doi: 10.1073/pnas.212516099, PMID: 12370419 PMC129730

[5] Chini EN Chini CC Nin V Escande C . Deleted in breast cancer-1 (Dbc-1) in the interface between metabolism, aging and cancer. Biosci Rep. (2013) 33:637–43. doi: 10.1042/bsr20130062, PMID: 23841676 PMC3755336

[6] Kim HJ Moon SJ Kim JH . Mechanistic insights into the dual role of ccar2/dbc1 in cancer. Exp Mol Med. (2023) 55:1691–701. doi: 10.1038/s12276-023-01058-1, PMID: 37524873 PMC10474295

[7] Zhao W Kruse JP Tang Y Jung SY Qin J Gu W . Negative regulation of the deacetylase sirt1 by dbc1. Nature. (2008) 451:587–90. doi: 10.1038/nature06515, PMID: 18235502 PMC2866287

[8] Rasha F Mims BM Castro-Piedras I Barnes BJ Grisham MB Rahman RL . The versatility of sirtuin-1 in endocrinology and immunology. Front Cell Dev Biol. (2020) 8:589016. doi: 10.3389/fcell.2020.589016, PMID: 33330467 PMC7717970

[9] Magni M Buscemi G Zannini L . Cell cycle and apoptosis regulator 2 at the interface between DNA damage response and cell physiology. Mutat Res Rev Mutat Res. (2018) 776:1–9. doi: 10.1016/j.mrrev.2018.03.004, PMID: 29807573

[10] Huang YH Yeh CT . Functional compartmentalization of hsp60-survivin interaction between mitochondria and cytosol in cancer cells. Cells. (2019) 9. doi: 10.3390/cells9010023, PMID: 31861751 PMC7016642

[11] Cha EJ Noh SJ Kwon KS Kim CY Park BH Park HS . Expression of dbc1 and sirt1 is associated with poor prognosis of gastric carcinoma. Clin Cancer Res. (2009) 15:4453–9. doi: 10.1158/1078-0432.Ccr-08-3329, PMID: 19509139

[12] Wang Z-P Hang L . Clinical significance of expression of deleted in breast cancer-1 in human gastric cancer. World Chin J Digestology. (2018) 26:150–8. doi: 10.11569/wcjd.v26.i3.150

[13] Huan Y Wu D Zhou D Sun B Li G . Dbc1 promotes anoikis resistance of gastric cancer cells by regulating nf-κb activity. Oncol Rep. (2015) 34:843–9. doi: 10.3892/or.2015.4007, PMID: 26035299

[14] Lee H Kim KR Noh SJ Park HS Kwon KS Park BH . Expression of dbc1 and sirt1 is associated with poor prognosis for breast carcinoma. Hum Pathol. (2011) 42:204–13. doi: 10.1016/j.humpath.2010.05.023, PMID: 21056897

[15] Sung JY Kim R Kim JE Lee J . Balance between sirt1 and dbc1 expression is lost in breast cancer. Cancer Sci. (2010) 101:1738–44. doi: 10.1111/j.1349-7006.2010.01573x, PMID: 20412117 PMC11159965

[16] Kim JR Moon YJ Kwon KS Bae JS Wagle S Yu TK . Expression of sirt1 and dbc1 is associated with poor prognosis of soft tissue sarcomas. PloS One. (2013) 8:e74738. doi: 10.1371/journal.pone.0074738, PMID: 24019980 PMC3760851

[17] Zhang Y Gu Y Sha S Kong X Zhu H Xu B . Dbc1 is over-expressed and associated with poor prognosis in colorectal cancer. Int J Clin Oncol. (2014) 19:106–12. doi: 10.1007/s10147-012-0506-5, PMID: 23299276

[18] Zhang Y Liu J Deng H Shao X Li H Guo X . Expression of DBC1 and its clinical significance in human colon cancer. Modern Oncol. (2016) 24:1776–9. doi: 10.3969/j.issn.1672-4992.2016.11.029

[19] Cho D Park H Park SH Kim K Chung M Moon W . The expression of dbc1/ccar2 is associated with poor prognosis of ovarian carcinoma. J Ovarian Res. (2015) 8:2. doi: 10.1186/s13048-015-0129-3, PMID: 25823848 PMC4335761

[20] Zhang S Liu Z . Expression of DBC1 in small cell lung cancer tissues and correlation with prognosis. J China Med Univ. (2018) 47:222–5. doi: 10.12007/j.issn.0258-4646.2018.03.007

[21] Wang L . Expression and clinical significance of DBC-1 in small cell lung cancer. Pract J Cancer. (2021) 36:1414–6. doi: 10.3969/j.issn.1001-5930.2021.09.006

[22] Kang Y Jung WY Lee H Lee E Kim A Kim BH . Expression of sirt1 and dbc1 in gastric adenocarcinoma. Korean J Pathol. (2012) 46:523–31. doi: 10.4132/KoreanJPathol.2012.46.6.523, PMID: 23323102 PMC3540329

[23] Noguchi A Kikuchi K Zheng H Takahashi H Miyagi Y Aoki I . Sirt1 expression is associated with a poor prognosis, whereas dbc1 is associated with favorable outcomes in gastric cancer. Cancer Med. (2014) 3:1553–61. doi: 10.1002/cam4.310, PMID: 25146318 PMC4298382

[24] Liu J Meng C Li C Tang K Tang H Liao J . Deleted in breast cancer 1 as a novel prognostic biomarker for digestive system cancers: A meta-analysis. J Cancer. (2019) 10:1633–41. doi: 10.7150/jca.26935, PMID: 31205519 PMC6548013

[25] Liu G Wu Q Wang Y Xiong Q Fu F . Deleted in breast cancer 1 as a potential prognostic biomarker in human cancers: A pooled analysis of 2,254 patients. Onco Targets Ther. (2019) 12:1563–74. doi: 10.2147/ott.S189618, PMID: 30863120 PMC6390861

[26] Page MJ McKenzie JE Bossuyt PM Boutron I Hoffmann TC Mulrow CD . The prisma 2020 statement: an updated guideline for reporting systematic reviews. Bmj. (2021) 372:n71. doi: 10.1136/bmj.n71, PMID: 33782057 PMC8005924

[27] Stang A . Critical evaluation of the newcastle-ottawa scale for the assessment of the quality of nonrandomized studies in meta-analyses. Eur J Epidemiol. (2010) 25:603–5. doi: 10.1007/s10654-010-9491-z, PMID: 20652370

[28] Higgins JP Thompson SG Deeks JJ Altman DG . Measuring inconsistency in meta-analyses. Bmj. (2003) 327:557–60. doi: 10.1136/bmj.327.7414.557, PMID: 12958120 PMC192859

[29] Stuck AE Rubenstein LZ Wieland D . Bias in meta-analysis detected by a simple, graphical test. Asymmetry detected in funnel plot was probably due to true heterogeneity. Bmj. (1998) 316:469; author reply 70–1. doi: 10.1136/bmj.316.7129.469, PMID: 9492685 PMC2665578

[30] Jung W Hong KD Jung WY Lee E Shin BK Kim HK . Sirt1 expression is associated with good prognosis in colorectal cancer. Korean J Pathol. (2013) 47:332–9. doi: 10.4132/KoreanJPathol.2013.47.4.332, PMID: 24009628 PMC3759632

[31] Ha SY Kim JH Yang JW Bae H Cho HY Park CK . Expression of dbc1 is associated with poor prognosis in hepatitis virus-related hepatocellular carcinoma. Pathol Res Pract. (2016) 212:616–21. doi: 10.1016/j.prp.2016.04.001, PMID: 27083241

[32] Li C Liao J Wu S Fan J Peng Z Wang Z . Overexpression of dbc1, correlated with poor prognosis, is a potential therapeutic target for hepatocellular carcinoma. Biochem Biophys Res Commun. (2017) 494:511–7. doi: 10.1016/j.bbrc.2017.10.134, PMID: 29106957

[33] Duan W Gu Y Yang Y Ma Y-Z Da C-X Wu X-H . Clinicopathological significance of the expression of DBC1 protein in human hepatocellular carcinoma tissue. Chin J Clin. (2012) 6:2370–3. doi: 10.3877/cma.j.issn.1674-0785.2012.09.075

[34] Kim SH Kim JH Yu EJ Lee KW Park CK . The overexpression of dbc1 in esophageal squamous cell carcinoma correlates with poor prognosis. Histol Histopathol. (2012) 27:49–58. doi: 10.14670/hh-27.49, PMID: 22127596

[35] Noh SJ Kang MJ Kim KM Bae JS Park HS Moon WS . Acetylation status of P53 and the expression of dbc1, sirt1, and androgen receptor are associated with survival in clear cell renal cell carcinoma patients. Pathology. (2013) 45:574–80. doi: 10.1097/PAT.0b013e3283652c7a, PMID: 24018803

[36] Park HS Bae JS Noh SJ Kim KM Lee H Moon WS . Expression of dbc1 and androgen receptor predict poor prognosis in diffuse large B cell lymphoma. Transl Oncol. (2013) 6:370–81. doi: 10.1593/tlo.13250, PMID: 23730418 PMC3660807

[37] Wagle S Park SH Kim KM Moon YJ Bae JS Kwon KS . Dbc1/ccar2 is involved in the stabilization of androgen receptor and the progression of osteosarcoma. Sci Rep. (2015) 5:13144. doi: 10.1038/srep13144, PMID: 26249023 PMC4642542

[38] Won KY Cho H Kim GY Lim SJ Bae GE Lim JU . High dbc1 (Ccar2) expression in gallbladder carcinoma is associated with favorable clinicopathological factors. Int J Clin Exp Pathol. (2015) 8:11440–5., PMID: 26617872 PMC4637688

[39] Yu XM Liu Y Jin T Liu J Wang J Ma C . The expression of sirt1 and dbc1 in laryngeal and hypopharyngeal carcinomas. PloS One. (2013) 8:e66975. doi: 10.1371/journal.pone.0066975, PMID: 23805287 PMC3689713

[40] Zhou L-B Jin M-M Guo S . Expression of DBC1 and p53 in papillary thyroid carcinoma and its clinical significance. Chin J Health Lab Tec. (2021) 31:358–61.

[41] Zhao G Zou C Song D Zhao S . Protein expression of deleted in breast cancer 1 and silent information regulator 1 in endometrial carcinoma and related clinical significance. J Qingdao Univ (medical sciences). (2022) 58:915–9. doi: 10.11721/jms.2096-5532.2022.58.193

[42] Fang Q Bellanti JA Zheng SG . Advances on the role of the deleted in breast cancer (Dbc1) in cancer and autoimmune diseases. J Leukoc Biol. (2021) 109:449–54. doi: 10.1002/jlb.6mr0320-086r, PMID: 32337788

[43] Kang H Suh JY Jung YS Jung JW Kim MK Chung JH . Peptide switch is essential for sirt1 deacetylase activity. Mol Cell. (2011) 44:203–13. doi: 10.1016/j.molcel.2011.07.038, PMID: 22017869 PMC3240942

[44] Wu QJ Zhang TN Chen HH Yu XF Lv JL Liu YY . The sirtuin family in health and disease. Signal Transduct Target Ther. (2022) 7:402. doi: 10.1038/s41392-022-01257-8, PMID: 36581622 PMC9797940

[45] Chang HC Guarente L . Sirt1 and other sirtuins in metabolism. Trends Endocrinol Metab. (2014) 25:138–45. doi: 10.1016/j.tem.2013.12.001, PMID: 24388149 PMC3943707

[46] Qin B Minter-Dykhouse K Yu J Zhang J Liu T Zhang H . Dbc1 functions as a tumor suppressor by regulating P53 stability. Cell Rep. (2015) 10:1324–34. doi: 10.1016/j.celrep.2015.01.066, PMID: 25732823 PMC4351187

[47] Akande OE Damle PK Pop M Sherman NE Szomju BB Litovchick LV . Dbc1 regulates P53 stability via inhibition of cbp-dependent P53 polyubiquitination. Cell Rep. (2019) 26:3323–5e4. doi: 10.1016/j.celrep.2019.02.076, PMID: 30893604 PMC6478392

[48] Menssen A Hydbring P Kapelle K Vervoorts J Diebold J Lüscher B . The C-myc oncoprotein, the nampt enzyme, the sirt1-inhibitor dbc1, and the sirt1 deacetylase form a positive feedback loop. Proc Natl Acad Sci U.S.A. (2012) 109:E187–96. doi: 10.1073/pnas.1105304109, PMID: 22190494 PMC3268300

[49] Kandoth C McLellan MD Vandin F Ye K Niu B Lu C . Mutational landscape and significance across 12 major cancer types. Nature. (2013) 502:333–9. doi: 10.1038/nature12634, PMID: 24132290 PMC3927368

[50] Chen X Zhang T Su W Dou Z Zhao D Jin X . Mutant P53 in cancer: from molecular mechanism to therapeutic modulation. Cell Death Dis. (2022) 13:974. doi: 10.1038/s41419-022-05408-1, PMID: 36400749 PMC9674619

[51] Ashton AW Dhanjal HK Rossner B Mahmood H Patel VI Nadim M . Acetylation of nuclear receptors in health and disease: an update. FEBS J. (2024) 291:217–36. doi: 10.1111/febs.16695, PMID: 36471658

[52] Kim HJ Kim SH Yu EJ Seo WY Kim JH . A positive role of dbc1 in pea3-mediated progression of estrogen receptor-negative breast cancer. Oncogene. (2015) 34:4500–8. doi: 10.1038/onc.2014.381, PMID: 25417701

[53] Kim HJ Moon SJ Kim SH Heo K Kim JH . Dbc1 regulates wnt/β-catenin-mediated expression of macc1, a key regulator of cancer progression, in colon cancer. Cell Death Dis. (2018) 9:831. doi: 10.1038/s41419-018-0899-9, PMID: 30082743 PMC6079074

[54] Yu EJ Kim SH Kim HJ Heo K Ou CY Stallcup MR . Positive regulation of β-catenin-prox1 signaling axis by dbc1 in colon cancer progression. Oncogene. (2016) 35:3410–8. doi: 10.1038/onc.2015.401, PMID: 26477307 PMC5058359

[55] Moon SJ Jeong BC Kim HJ Lim JE Kwon GY Kim JH . Dbc1 promotes castration-resistant prostate cancer by positively regulating DNA binding and stability of ar-V7. Oncogene. (2018) 37:1326–39. doi: 10.1038/s41388-017-0047-5, PMID: 29249800

[56] Kim HJ Moon SJ Hong S Won HH Kim JH . Dbc1 is a key positive regulator of enhancer epigenomic writers kmt2d and P300. Nucleic Acids Res. (2022) 50:7873–88. doi: 10.1093/nar/gkac585, PMID: 35801925 PMC9371912

[57] Liu Q Luo Q Feng J Zhao Y Ma B Cheng H . Hypoxia-induced proteasomal degradation of dbc1 by siah2 in breast cancer progression. Elife. (2022) 11. doi: 10.7554/eLife.81247, PMID: 35913115 PMC9377797

