# Supplementary figures and images for: Prognostic and clinicopathological value of dbc1 expression in human cancers: a systematic review and meta-analysis
Source: Front Oncol. 2025 Jul 7;15:1584622. doi: 10.3389/fonc.2025.1584622 (PMC12278428; doi:10.3389/fonc.2025.1584622)

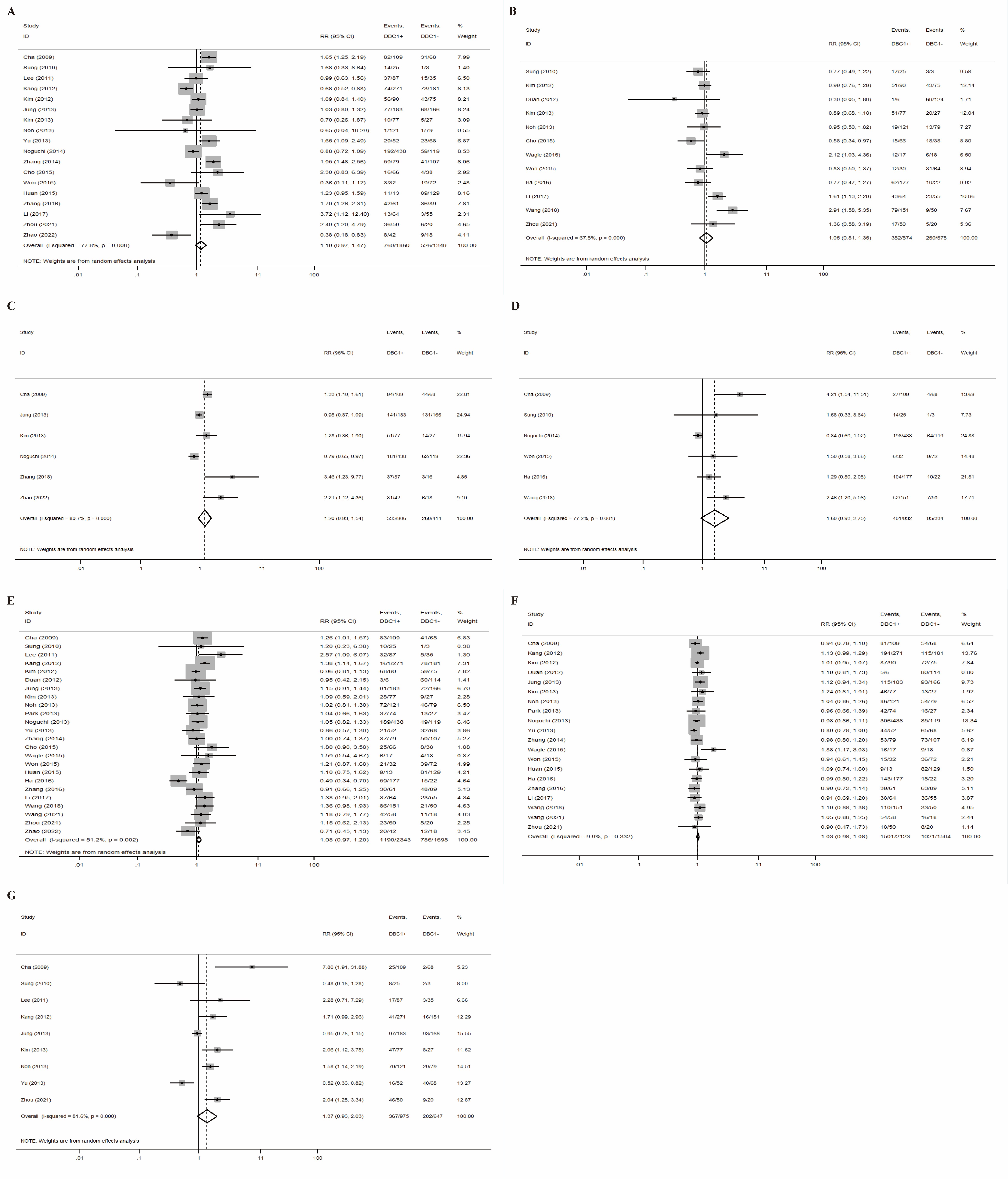

Supplement: Supplementary Figure 1 — Forest plot of DBC1 expression and other clinicopathological characteristics in various cancers. [file Image1.jpeg]

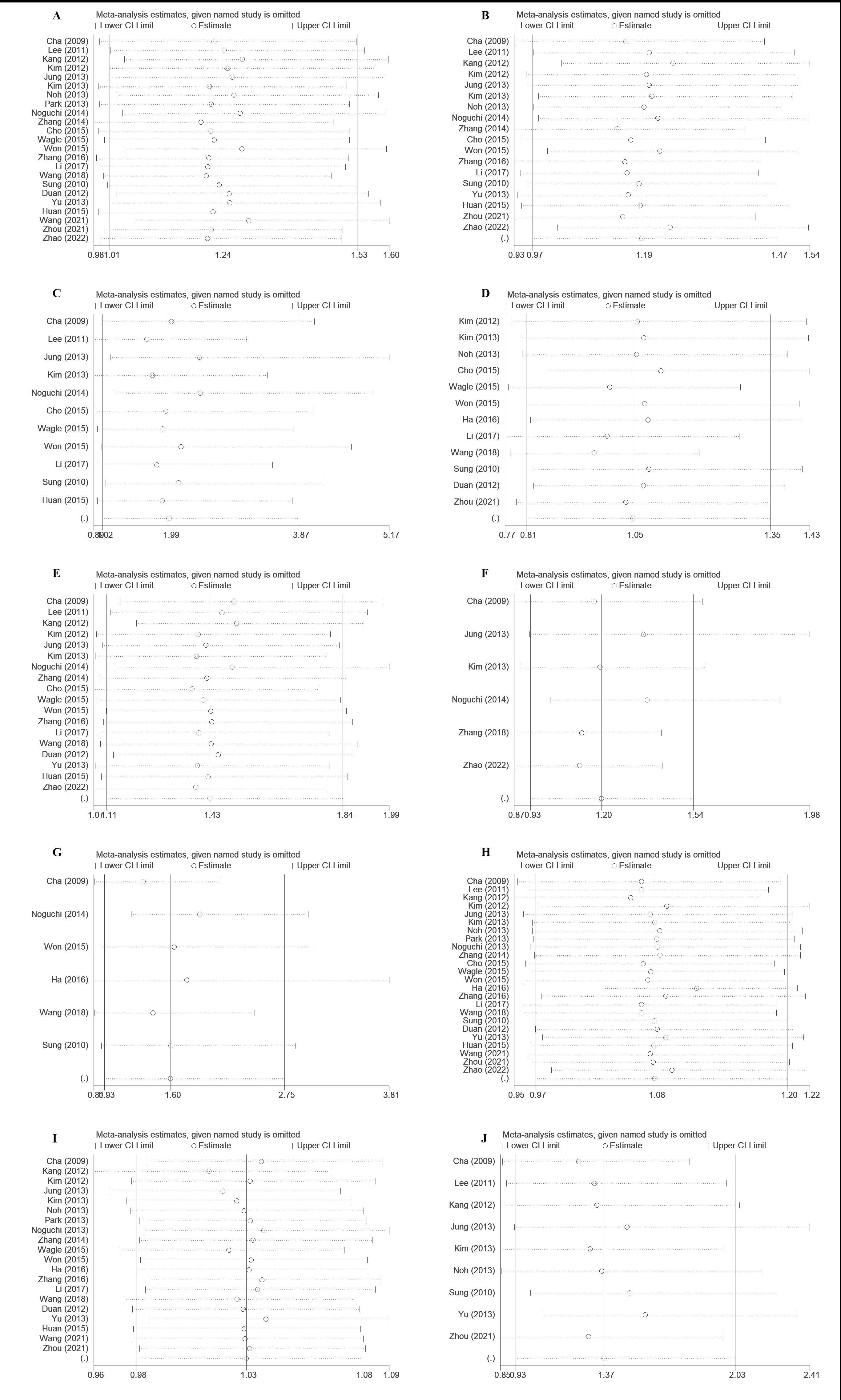

Supplement: Supplementary Figure 2 — Sensitive analysis of clinicopathological characteristics for patients in various cancers. [file Image2.jpeg]

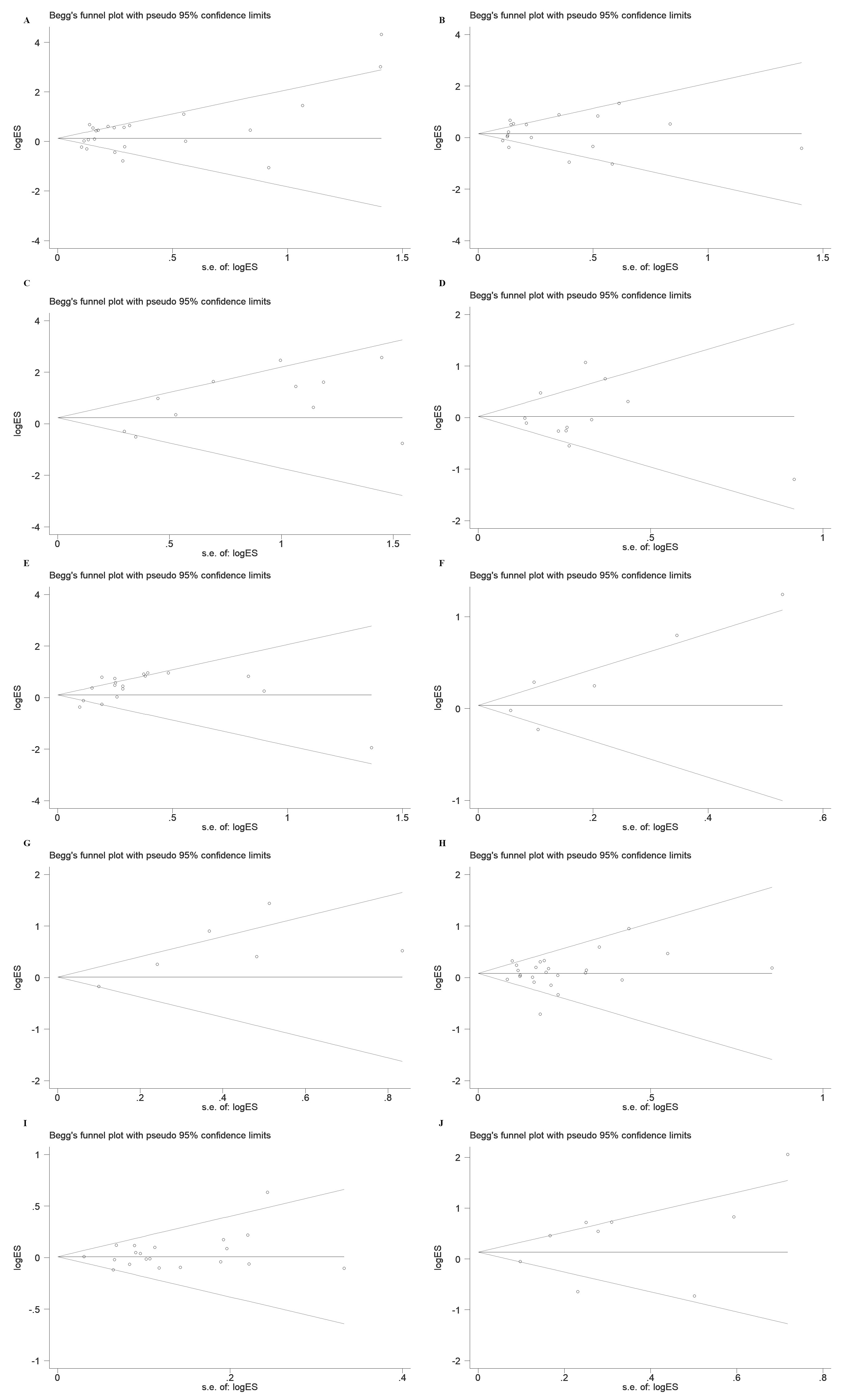

Supplement: Supplementary Figure 3 — Begg’s funnel plots of publication bias for clinicopathological characteristics in various cancers. [file Image3.jpeg]
