# Supplementary material for: Prognostic and clinicopathological value of dbc1 expression in human cancers: a systematic review and meta-analysis
Source: Front Oncol. 2025 Jul 7;15:1584622. doi: 10.3389/fonc.2025.1584622 (PMC12278428; doi:10.3389/fonc.2025.1584622)
Supplement: Supplementary file 4 [file Table1.docx]

**Search Strategy**

**Search included: Pubmed, Web of Science, Embase, CNKI, Wanfang**

**Publication date to 2023/5/11**

| **Database** | **Search strategy** |
| --- | --- |
| Pubmed | ("CCAR2 protein, human" [Supplementary Concept]) OR ((((((((((((((((DBC1[Title/Abstract]) OR (KIAA1967[Title/Abstract])) OR ("deleted in breast cancer 1"[Title/Abstract])) OR ("Deleted in Breast Cancer-1"[Title/Abstract])) OR ("cell cycle and apoptosis regulator 2"[Title/Abstract])) OR ("deleted in breast cancer gene 1"[Title/Abstract])) OR (CCAR2[Title/Abstract])) OR (DBC-1[Title/Abstract])) OR (NET35[Title/Abstract])) OR ("Cell Division Cycle And Apoptosis Regulator Protein 2"[Title/Abstract])) OR ("Cell Cycle And Apoptosis Regulator Protein 2"[Title/Abstract])) OR ("P30 DBC"[Title/Abstract])) OR ("Deleted In Breast Cancer Gene 1 Protein"[Title/Abstract])) OR ("DBIRD Complex Subunit KIAA1967"[Title/Abstract])) OR (P30DBC[Title/Abstract])) OR (DBC.1[Title/Abstract])) |
| WOS | ((((((((((((((((TS=("CCAR2 protein")) OR TS=(DBC1)) OR TS=(KIAA1967)) OR TS=("deleted in breast cancer 1")) OR TS=("Deleted in Breast Cancer-1")) OR TS=("cell cycle and apoptosis regulator 2")) OR TS=("deleted in breast cancer gene 1")) OR TS=(CCAR2)) OR TS=(DBC-1)) OR TS=(NET35)) OR TS=("Cell Division Cycle And Apoptosis Regulator Protein 2")) OR TS=("Cell Cycle And Apoptosis Regulator Protein 2")) OR TS=("P30 DBC")) OR TS=("Deleted In Breast Cancer Gene 1 Protein")) OR TS=("DBIRD Complex Subunit KIAA1967")) OR TS=(P30DBC)) OR TS=(DBC.1) |
| Embase | 'dbc1 gene'/exp OR 'dbc1 protein'/exp OR 'ccar2 protein':ti,ab OR dbc1:ti,ab OR kiaa1967:ti,ab OR 'deleted in breast cancer 1':ti,ab OR 'deleted in breast cancer-1':ti,ab OR 'cell cycle and apoptosis regulator 2':ti,ab OR 'deleted in breast cancer gene 1':ti,ab OR ccar2:ti,ab OR 'dbc 1':ti,ab OR net35:ti,ab OR 'cell division cycle and apoptosis regulator protein 2':ti,ab OR 'cell cycle and apoptosis regulator protein 2':ti,ab OR 'p30 dbc':ti,ab OR 'deleted in breast cancer gene 1 protein':ti,ab OR 'dbird complex subunit kiaa1967':ti,ab OR p30dbc:ti,ab OR dbc.1:ti,ab |
| CNKI | TKA='DBC1'+'KIAA1967'+'deleted in breast cancer 1'+'Deleted in Breast Cancer-1'+'cell cycle and apoptosis regulator 2'+'deleted in breast cancer gene 1'+'CCAR2'+'DBC-1'+'NET35'+'Cell Division Cycle And Apoptosis Regulator Protein 2'+'Cell Cycle And Apoptosis Regulator Protein 2'+'P30 DBC'+'Deleted In Breast Cancer Gene 1 Protein'+'DBIRD Complex Subunit KIAA1967'+'P30DBC'+'DBC.1'+'乳腺癌缺失基因1'+'乳腺癌缺失蛋白1'+'细胞周期和细胞凋亡调节因子2'+'细胞周期和细胞凋亡调节蛋白2' |
| Wanfang | 主题:"DBC1"or"KIAA1967"or"deleted in breast cancer 1"or"Deleted in Breast Cancer-1"or"cell cycle and apoptosis regulator 2"or"deleted in breast cancer gene 1"or"CCAR2"or"DBC-1"or"NET35"or"Cell Division Cycle And Apoptosis Regulator Protein 2"or"Cell Cycle And Apoptosis Regulator Protein 2"or"P30 DBC”or”Deleted In Breast Cancer Gene 1 Protein"or"DBIRD Complex Subunit KIAA1967"or"P30DBC"or"DBC.1"or"乳腺癌缺失基因1"or"乳腺癌缺失蛋白1"or"细胞周期和细胞凋亡调节因子2"or"细胞周期和细胞凋亡调节蛋白2" |
