# Supplementary material for: Prognostic and clinicopathological value of dbc1 expression in human cancers: a systematic review and meta-analysis
Source: Front Oncol. 2025 Jul 7;15:1584622. doi: 10.3389/fonc.2025.1584622 (PMC12278428; doi:10.3389/fonc.2025.1584622)
Supplement: Supplementary file 6 [file Table3.docx]

**Table S3. Meta regression analysis**

| **Covariate** | **OS (n=17)** | | | **RFS (n=11)** | | |
| --- | --- | --- | --- | --- | --- | --- |
|  | β | 95% CI | *P* | β | 95% CI | *P* |
| **Cancer type** | 0.38 | (−0.07–0.82) | 0.090 | 0.19 | (−0.14–0.53) | 0.198 |
| **Cancer** | **−0.13** | **(−0.27–−0.004)** | **0.044** | −0.07 | (−0.20–0.06) | 0.208 |
| **Country** | −0.21 | (−1.12–0.69) | 0.617 | **−0.99** | **(−1.67–−0.31)** | **0.013** |
| **Sample size** | **−1.10** | **(−2.12–−0.07)** | **0.038** | −0.71 | (−1.46–0.04) | 0.06 |
| **Cutoff value** | −0.13 | (−0.52–0.26) | 0.479 | 0.10 | (−0.22–0.43) | 0.461 |
| **Adj R² (%)** | 58.85 | | | 100 | | |
| **I²_res (%)** | 74.72 | | | 0 | | |
| **Model P** | 0.024 | | | 0.004 | | |
